# Cryo-EM structure of the fully assembled Elongator complex

**DOI:** 10.1093/nar/gkac1232

**Published:** 2023-01-09

**Authors:** Marcin Jaciuk, David Scherf, Karol Kaszuba, Monika Gaik, Alexander Rau, Anna Kościelniak, Rościsław Krutyhołowa, Michał Rawski, Paulina Indyka, Andrea Graziadei, Andrzej Chramiec-Głąbik, Anna Biela, Dominika Dobosz, Ting-Yu Lin, Nour-el-Hana Abbassi, Alexander Hammermeister, Juri Rappsilber, Jan Kosinski, Raffael Schaffrath, Sebastian Glatt

**Affiliations:** Malopolska Centre of Biotechnology (MCB), Jagiellonian University, Krakow 30-387, Poland; Institute for Biology, Department for Microbiology, University of Kassel, Kassel 34132, Germany; European Molecular Biology Laboratory Hamburg, Hamburg 22607, Germany; Centre for Structural Systems Biology (CSSB), Hamburg 22607, Germany; Malopolska Centre of Biotechnology (MCB), Jagiellonian University, Krakow 30-387, Poland; Bioanalytics, Institute of Biotechnology, Technische Universität Berlin, Berlin 13355, Germany; Malopolska Centre of Biotechnology (MCB), Jagiellonian University, Krakow 30-387, Poland; Malopolska Centre of Biotechnology (MCB), Jagiellonian University, Krakow 30-387, Poland; Malopolska Centre of Biotechnology (MCB), Jagiellonian University, Krakow 30-387, Poland; National Synchrotron Radiation Centre SOLARIS, Jagiellonian University, Krakow 30-387, Poland; Malopolska Centre of Biotechnology (MCB), Jagiellonian University, Krakow 30-387, Poland; National Synchrotron Radiation Centre SOLARIS, Jagiellonian University, Krakow 30-387, Poland; Bioanalytics, Institute of Biotechnology, Technische Universität Berlin, Berlin 13355, Germany; Malopolska Centre of Biotechnology (MCB), Jagiellonian University, Krakow 30-387, Poland; Malopolska Centre of Biotechnology (MCB), Jagiellonian University, Krakow 30-387, Poland; Malopolska Centre of Biotechnology (MCB), Jagiellonian University, Krakow 30-387, Poland; Malopolska Centre of Biotechnology (MCB), Jagiellonian University, Krakow 30-387, Poland; Malopolska Centre of Biotechnology (MCB), Jagiellonian University, Krakow 30-387, Poland; Postgraduate School of Molecular Medicine, Medical University of Warsaw, Warsaw 02-091, Poland; Malopolska Centre of Biotechnology (MCB), Jagiellonian University, Krakow 30-387, Poland; Institute for Biology, Department for Microbiology, University of Kassel, Kassel 34132, Germany; Bioanalytics, Institute of Biotechnology, Technische Universität Berlin, Berlin 13355, Germany; Wellcome Centre for Cell Biology, University of Edinburgh, Edinburgh EH9 3BF, UK; European Molecular Biology Laboratory Hamburg, Hamburg 22607, Germany; Centre for Structural Systems Biology (CSSB), Hamburg 22607, Germany; Structural and Computational Biology Unit, European Molecular Biology Laboratory, Heidelberg 69117, Germany; Institute for Biology, Department for Microbiology, University of Kassel, Kassel 34132, Germany; Malopolska Centre of Biotechnology (MCB), Jagiellonian University, Krakow 30-387, Poland

## Abstract

Transfer RNA (tRNA) molecules are essential to decode messenger RNA codons during protein synthesis. All known tRNAs are heavily modified at multiple positions through post-transcriptional addition of chemical groups. Modifications in the tRNA anticodons are directly influencing ribosome decoding and dynamics during translation elongation and are crucial for maintaining proteome integrity. In eukaryotes, wobble uridines are modified by Elongator, a large and highly conserved macromolecular complex. Elongator consists of two subcomplexes, namely Elp123 containing the enzymatically active Elp3 subunit and the associated Elp456 hetero-hexamer. The structure of the fully assembled complex and the function of the Elp456 subcomplex have remained elusive. Here, we show the cryo-electron microscopy structure of yeast Elongator at an overall resolution of 4.3 Å. We validate the obtained structure by complementary mutational analyses *in vitro* and *in vivo*. In addition, we determined various structures of the murine Elongator complex, including the fully assembled mouse Elongator complex at 5.9 Å resolution. Our results confirm the structural conservation of Elongator and its intermediates among eukaryotes. Furthermore, we complement our analyses with the biochemical characterization of the assembled human Elongator. Our results provide the molecular basis for the assembly of Elongator and its tRNA modification activity in eukaryotes.

## Graphical Abstract

**Graphical Abstract:**
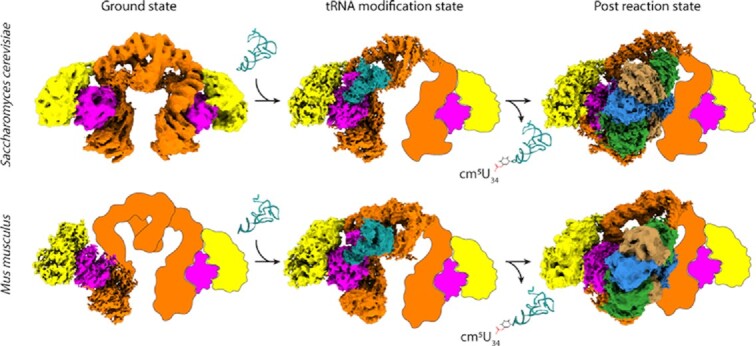
The Elp123 subcomplex of Elongator captures tRNA substrates and modifies them, whereas the Elp456 assists with the release of the modified tRNAs. Yeast and mouse Elongator are structurally almost identical.

## INTRODUCTION

Transfer RNAs (tRNA) are one of the most essential biomolecules in all living organisms. Together with messenger RNAs (mRNA), they are responsible for the correct transfer of information from the genomic DNA into functional polypeptide chains. tRNAs undergo significant post-transcriptional modifications, which confer stabilization of the folded tRNA, regulate the interaction with other molecules or tune the codon-anticodon pairing during the mRNA translation process within the ribosome (1). In eukaryotes, almost all wobble uridines at position 34 (U_34_), are carboxymethylated on the fifth carbon of their base (cm^5^) by the activity of the highly conserved Elongator complex aided by its accessory proteins Kti11, Kti12, Kti13, Kti14 and Sit4 (2–6). Subsequently, cm^5^ is further modified by other enzymes to 5-methoxycarbonylmethyl (mcm^5^), 5-carbamoylmethyl (ncm^5^) or 5-methoxy-carbonyl-methyl-2-thio (mcm^5^s^2^) (7,8). These modifications regulate proper tRNA binding in the A-site of translating ribosomes during the elongation phase (9–11) and thus maintain an optimal speed during polypeptide synthesis, supporting proper co-translational folding of the nascent proteins. Decreased levels of wobble uridine modifications lead to ribosome pausing, incorrect protein folding and consequently trigger intracellular protein aggregation and proteotoxic stress (12–14). Furthermore, Elongator is a clinically highly relevant enzyme, as mutations that disturb the structure or function of the complex result in severe human diseases (15), like familial dysautonomia (16), rolandic epilepsy (17), intellectual disability (18), amyotrophic lateral sclerosis (19), other neurodevelopmental diseases (20–22) and childhood medulloblastoma (23).

The Elongator complex harbors two copies of each of its six subunits, namely Elongator proteins 1–6 (Elp1–Elp6) (24). The complex can be divided into two discrete subcomplexes - the larger catalytic Elp123 and the smaller hexameric ring-shaped Elp456 (25,26). Studies applying negative stain electron microscopy, revealed that *Saccharomyces cerevisiae* Elp123 (*Sc*Elp123) adopts a symmetric ‘moth’-like shape, with two distinguishable lobes that are separated by a cleft and connected by an arch region. In contrast, the structure of the fully assembled Elongator complex seems asymmetric as Elp456 only binds to one lobe of the dimeric Elp123 subcomplex (27,28). Based on these initial low-resolution reconstructions the following subunit arrangement of the Elp123 subcomplex was proposed – the two N-terminal WD40 domains of Elp1 and Elp2 (29), encompass the enzymatically active Elp3 subunit from opposite directions creating a lobe of Elp123. The remaining C-terminal domain (CTD) of Elp1 consisting of tetratricopeptide repeats (TPRs) and the CTD of the second copy of Elp1 dimerize into an arch-like structure that connects two Elp123 lobes, respectively (30). Subsequent single particle cryo-electron microscopy (cryo-EM) studies resulted in high-resolution structures of yeast Elp123 in its free and tRNA-bound states. Intriguingly, these structures revealed the spatial organization of the active site in the eukaryotic Elp3 protein, including the coordination of an iron-sulfur cluster, the *S*-adenosyl-methionine (SAM) cleavage product 5′-deoxyadenosine (5′-dA) and the respective tRNA substrate (31). Of note, the structure of yeast Elp3 resembles previously resolved crystal structures of bacterial and archaeal Elp3 proteins (32,33). In detail, the Elp3 subunit specifically binds the tRNA anticodon stem loop (ASL) in the vicinity of its radical *S*-adenosyl methionine (rSAM) and lysine acetyltransferase (KAT) domains to modify the well-positioned U_34_ nucleotide (32–34). tRNA binding is aided by additional contacts in the CTD of Elp1, which was previously shown to be heavily phosphorylated (35) and involved in tRNA binding (36).

Despite all previous efforts, several fundamental questions concerning Elongator's function remain elusive. For instance, the tRNA modification reaction has not been reconstituted *in vitro* (37,38), which leaves the exact molecular mechanisms and the observed substrate specificity unanswered. One of the main open questions concerns the functional role of the Elp456 subcomplex, which interacts with Elp123 and is essential for the activity of Elongator in yeast (39). To address these outstanding questions, we investigated the cryo-EM structures of the yeast Elongator complex in its native and bis(sulfosuccinimidyl)suberate (BS3) stabilized state. Next, we validated the functional relevance of the newly identified structural features *in vivo* using established phenotypic assays. Furthermore, we were able to confirm the high structural conservation of Elongator and its active site by determining structures of Elp123, Elp123–tRNA and Elongator complexes from yeast and mouse. Finally, through biochemical characterization of human Elp123 and Elp456 we can propose a detailed reaction mechanism and suggest a role for the Elp456 subcomplex during the reaction cycle of the Elongator.

## MATERIALS AND METHODS

### Preparation of expression constructs

The ORFs of *Saccharomyces cerevisiae ELP1* to *ELP6* were amplified by polymerase chain reactions (PCRs) and cloned in pairs into three individual vectors with inducible *GAL1* and *GAL10* promoters to allow for simultaneous expression of two gene products from a single vector (40). Yeast cells co-transformed with all three constructs, were selected for tryptophan, leucine and uracil prototrophy. Coding sequences for FLAG and Twin-Strep-tag (IBA Lifesciences) were added in frame to *ELP1* and *ELP3*, respectively. In addition, Elp6 was overproduced with a *Staphylococcus aureus* protein A tag and a tobacco etch virus (TEV) protease cleavage site to facilitate the purification from yeast cells. All sequences were introduced at the 3′ end of the individual ORFs. *Mus musculus* and *Homo sapiens* biGBac (41,42) constructs containing the ORFs of *ELP123* and *ELP456* were prepared using a similar tagging strategy. After PCR amplification all expression cassettes (except for *ELP2*) were cloned separately into pFastBac1 plasmids. *ELP3* was cloned in frame with a Twin-Strep-Tag sequence and *ELP6* with FLAG-TEV-Protein A sequence at their 3′ ends. *ELP2* genes were cloned into pFastBac-HTA with a 6xHis-tag sequence at its 5′ end. All genes were amplified from the pFastBac plasmids by PCR using primers with predefined 5′ and 3′ overhangs in each gene, followed by Gibson assembly reactions (43,44) (NEB) using pBig1a and pBig1b plasmids to obtain *ELP123* and *ELP456*, respectively (41). Mutagenesis in human *ELP1*, *ELP4* and *ELP5* were carried out using the QuikChange method. All genetic constructs were confirmed by DNA sequencing and moved to the insect cells expression system using standard Bac-to-Bac protocols.

### Protein production and purification

DS1-2b yeast cells (MAT α *his3-Δ200*, *leu2-Δ1*, *trp1-Δ63*, *ura3-52*), co-transformed with the constructs expressing all six Elongator genes, were grown on synthetic drop-out (SD) media plates (2% w/v agar and 2% w/v glucose) lacking tryptophan, leucine and uracil. Cells were grown shaking overnight at 30°C in the SD media (2% w/v glucose), reinoculated (3:50 ratio) on the next day into fresh SD medium and further incubated overnight at 30°C with shaking. For protein overproduction, cells were inoculated (1:20) in fresh SD media containing 2% w/v raffinose and incubated for 5–7 h at 30°C while shaking. *ELP* genes expression was induced by addition of galactose to a final concentration of 2% w/v and cells were left shaking for 21 h at 30°C. Cells were harvested by centrifugation, resuspended in lysis buffer (250 mM HEPES (pH 7.5), 100 mM NaCl, 10% v/v glycerol, 0.1% v/v Tween 20, 1 mM sodium orthovanadate, 20 mM sodium fluoride, aprotinin (2 μg/ml), leupeptin (5 μg/ml), 1 μM pepstatin A, and 1 mM PMSF) and small cell suspensions droplets were flash frozen in liquid nitrogen. Droplets were lysed by cryo-milling (Qiagen TissueLyser II) and stored as a powder at −80°C until use. All purification steps were performed at 4°C or on ice. 12 ml of fresh lysis buffer was added per 40 g of dry yeast pellet and thawed for 1 h under agitation. Lysates were clarified by two consecutive centrifugation steps at 21 000 × g for 30 min. Cell extracts were incubated for 30 min with 3 ml of IgG Sepharose™ 6 Fast Flow resin (GE Healthcare) and washed three times with lysis buffer. Proteins were eluted in 5 ml lysis buffer by TEV protease cleavage (1 mg) in presence of 1 mM dithiothreitol (DTT) for 1 h. Subsequently, the eluent was applied to a 1 ml StrepTrap HP column (GE Healthcare) and eluted with 20 mM d-desthiobiotin. The complex was concentrated using an Amicon Ultra-15 centrifugal filter (100 kDa cut-off) and the Elongator was further purified on a Superose 6 Increase 10/300 GL column (GE Healthcare) equilibrated with 20 mM HEPES (pH 7.5), 100 mM NaCl and 3 mM DTT. Selected fractions were pooled and concentrated with an Amicon Ultra-0.5 centrifugal filter (100 kDa cut-off). Aliquots were frozen in liquid nitrogen and stored at −80°C for further use.

Mouse and human Elp123 were overproduced in Super Sf9-1, Sf9-3 respectively, while both Elp456 in Hi-5 cells. Cells were cultured in ESF921 medium (Expression Systems) to a density of 800 000 cells/ml for Super Sf9-1/Sf9-3 and 500 000 cells/ml for Hi-5 cells, followed by baculovirus infection with the multiplicity of infection (MOI) of 1 and grown for 3 days at 27°C while shaking. Cell pellets were harvested and lysed in 50 mM HEPES (pH 7.5), 100 mM NaCl for Elp123 and 300 mM NaCl for Elp456, 2 mM MgCl_2_, 2 mM DTT, 5% v/v glycerol, aprotinin (2 μg/ml), leupeptin (5 μg/ml), 1 μM pepstatin A, 1 mM phenylmethylsulfonyl fluoride in presence of DNase I by three freeze-thaw cycles and sonication. Lysates were clarified by two consecutive centrifugation steps at 4°C and 63 000 × g for 30 and 60 min. Elp123 subcomplexes were purified from cell extracts using a 5 ml StrepTrap HP column (GE Healthcare) and eluted with 10 mM *d*-desthiobiotin. Next, Elp123 subcomplexes were further purified on a 5 ml HiTrap Heparin HP column (GE Healthcare) and eluted with a linear KCl gradient (50 mM HEPES pH 7.5, 1000 mM KCl, 1 mM DTT). The final purification step was carried out on a Superose 6 Increase 10/300 GL column (GE Healthcare) equilibrated with 20 mM HEPES (pH 7.5), 100 mM NaCl and 5 mM DTT. Mouse and human Elp456 subcomplexes were purified from cell extracts using IgG Sepharose™ 6 Fast Flow resin equilibrated with 50 mM HEPES (pH 7.5), 300 mM NaCl, 1 mM DTT and 5% v/v glycerol. Elution from the resin was accomplished by TEV protease cleavage (1 mg/5 ml). Eluates were concentrated using Amicon Ultra-15 centrifugal filters (100 kDa cut-off) and subjected to a Superose S6 Increase 10/300 GL column (GE Healthcare) equilibrated with 20 mM HEPES (pH 7.5), 100 mM NaCl and 5 mM DTT. Purity, integrity, protein quality and all complexes stoichiometry were monitored during every step by SDS-PAGE analysis and visualization with Coomassie stain.

### Bulk yeast tRNA isolation

To obtain yeast bulk tRNA, *S. cerevisiae* strains were grown in YPD (Yeast Extract–Peptone–Dextrose) to an OD_600 nm_ of 1, harvested, washed and resuspended in NucleoZOL (Machery Nagel). Cells were lysed by bead beating in 6 cycles each 60 s, 20% v/v volume of chloroform was added and samples were vigorously vortexed for another 60 s. Subsequently, the suspension was incubated for 15 min at 4°C and centrifugated for 30 min at 21 000 × g. The aqueous RNA-containing phase was transferred into a new reaction tube and large RNAs were precipitated for 3 h at −20°C using 2/3 volume of 8 M LiCl. Large RNAs were pelleted for 30 min at 4°C and 21 000 × g. To increase tRNA purity, the LiCl precipitation step was repeated. The tRNA-containing supernatant was precipitated with 10% v/v 3 M sodium acetate pH 5.2 and 2.5 volumes 100% ethanol overnight at −20°C. The tRNA containing pellet was washed twice with 75% v/v ethanol, air dried and resuspended in 10 mM sodium acetate pH 5.2. tRNA concentrations were determined using the Epoch (Agilent BioTek) spectrophotometer and stored at −80°C.

### Human bulk tRNA isolation

HEK293T cells cultured on 15-cm Petri dishes were washed with PBS and lysed for 5 min in 250 μl RNA lysis buffer (10 mM Tris–HCl (pH 7.5), 100 mM NaCl, 10 mM MgCl_2_, 1% TritonX-100 v/v, 0.5% v/v sodium deoxycholate, 0.5 mM DTT) at 4°C. The lysate was mixed with MiliQ water at a 1:1 ratio v/v and the RNA was isolated three times with 1 volume of acid-phenol:chloroform (1:1 ratio v/v), followed by an additional step using 1 volume chloroform. After each step the sample was vortexed and centrifugated for 10 min at 4500 × g at 4°C and the aqueous phases were collected, combined and mixed with 10% volume of 3 M sodium acetate pH 5.5, three volumes of 100% ethanol, 1 μl of glycogen and incubated overnight at −80°C. The precipitate, was centrifuged at 8000 × g for 30 min at 4°C and the supernatant was discarded. The pellet was resuspended in 100 μl equilibration buffer without TritonX-100 (10 mM Bis–Tris (pH 6.3), 15% ethanol v/v, 200 mM KCl). RNA concentration was measured using a NanoDrop spectrophotometer and the sample was stored at −80°C until use. To isolate bulk tRNA 900 μg of total RNA (diluted in 2 ml equilibration buffer without TritonX-100) was loaded on an equilibrated NucleoBond AX100 column (10 mM Bis–Tris (pH 6.3), 15% v/v ethanol, 200 mM KCl, 0.15% v/v TritonX-100) and washed twice with 12 ml washing buffer (10 mM Bis–Tris (pH 6.3), 15% v/v ethanol, 500 mM KCl) tRNA was eluted with 12 ml elution buffer (10 mM Bis–Tris (pH 6.3), 15% v/v ethanol, 800 mM KCl) and mixed with 2.5 volumes of 100% ethanol, 1 μl of glycogen and incubated overnight at −80°C. After precipitation, the sample was centrifuged for 30 min at 8000 × g (4°C), and the supernatant was discarded. The tRNA pellet was washed with 40 ml 80% v/v EtOH by vortexing. Afterward, the tRNA was centrifuged for 30 min at 8000 × g (4°C). Subsequently, the tRNA pellet was air dried and resuspended in 30 μl RNAse free water. tRNA concentration was measured using a NanoDrop spectrophotometer and the sample was stored at −80°C for later use.

### Electron microscopy

QUANTIFOIL^®^ R 2/1 copper grids (200 mesh) were glow-discharged on a Leica EM ACE 200 glow discharger (8 mA, 60 s). 2.5 μl of the sample at 0.4–0.6 mg/ml concentration was plunge-frozen using a Vitrobot Mark IV (Thermo Fisher) set to 100% humidity and 4°C with the following blotting parameters − 15 s wait time, blot force 5 and 5 s blot time. tRNA was used at 3–5 molar excess with 15 min incubation on ice for *Sc*Elp123–tRNA and 37°C for mouse Elp123 (*Mm*Elp123)–tRNA samples. *Sc*Elp123–tRNA micrographs were acquired at 300 kV using a Titan Krios 2 (FEI; EMBL, Germany) equipped with a Gatan Quantum energy filter and a K2 Summit direct electron detector. 11,080 micrographs were collected with 0.81 Å pixel size and 0.8–2 μm under-focus for a total of 40 frames accumulating 42.45 e^−^/Å^2^ dose. The remaining datasets were collected on Titan Krios G3i (Thermo Fisher; Solaris, Poland) equipped with a Gatan Quantum energy filter and a K3 Summit direct electron detector. The used under-focus range was 0.9–3 μm for a total of 40 frames accumulating an overall dose of 40 e^−^/Å^2^. 6339, 4716, 6415, 7000 and 5232 micrographs were collected for *Sc*Elongator, *Sc*Elp456, *Mm*Elp123, *Mm*Elp123–tRNA and *Mm*Elongator, respectively with a pixel size of 1.1 Å for *Sc*Elp456 and 0.86 Å for the remaining data sets.

### Image processing

Frame alignment and dose weighting were performed with Relion 3.1 (45) implementation of motion correction for *Sc*Elp123–tRNA, *Sc*Elp456 and *Mm*Elp123; with WARP 1.0.9 (46) for *Sc*Elongator, and cryoSPARC (47) for *Mm*Elp123–tRNA and *Mm*Elongator. Averaged micrographs for all datasets were imported into cryoSPARC and the contrast transfer function (CTF) was corrected. Blob picking was performed on 10–15% of micrographs to select an initial set of particles for template picking (*Mm*Elp123–tRNA) and to train the TOPAZ particle picker (48). For *Sc*Elp456 and *Mm*Elp123 two separate trainings were performed for different orientations. The resulting TOPAZ models were used next to pick particles from all micrographs of respective data sets. A similar approach was applied for the *Mm*Elongator dataset, where two TOPAZ picking jobs were used during the analysis. Extracted particles were binned as indicated in respective processing pipeline panels and particle curation was performed via 2D classification and *ab-initio* reconstructions. All Elongator-related particles, except for *Mm*Elp123–tRNA, were next converted with pyem (49) and imported into Relion for further sub-classification. For *Sc*Elongator and *Mm*Elongator, masked 3D classifications around Elp456 were applied to improve the densities of the smaller subcomplex. For *Sc*Elp123–tRNA masked tRNA 3D classification followed by CTF refinement and Bayesian polishing were performed to improve the tRNA density. For the *Mm*Elp123 active site, a masked 3D classification was performed that resulted in an improved density around the bound SAM molecule. The final maps after post-processing were obtained at the following resolutions − 4.35 Å for *Sc*Elongator, 3.96 Å for *Sc*Elp123–tRNA, 3.7 Å for *Sc*Elp456, 4.01 Å for *Mm*Elp123 and 5.92 Å for *Mm*Elongator, respectively. The analysis of *Mm*Elp123–tRNA was exclusively performed in cryoSPARC resulting in an overall resolution of 4.35 Å.

### Model building, refinement and validation

Atomic models for both, yeast and mouse structures, were prepared via SWISS-MODEL homology model building (50–58), using PDB entries 6QK7 and 4A8J as templates. The model of the yeast Elp3 subunit from Elp123–tRNA was replaced with the AlphaFold2 model (59) to include the previously missing N-terminus. After rigid body fitting into the cryo-EM density map, the models were manually curated and corrected in Coot (60) and further fitted by molecular dynamic flexible fit using MDFF (61) and Namdinator (62). The obtained atomic models were refined in Phenix (63,64), analyzed in Coot and validated with MolProbity (65) (Table 1). Figures were prepared using PyMOL (The PyMOL Molecular Graphics System, Version 2.5.1 Schrödinger, LLC) and UCSF ChimeraX (66).

**Table 1.** Cryo-EM data collection, refinement and validation statistics

	*Sc*Elongator EMD-15622 PDB ID 8ASV	*Sc*Elp456 EMD-15635 PDB ID 8AT6	*Sc*Elp123–tRNA EMD-15623 PDB ID 8ASW	*MmElp*123 EMD-15682 PDB ID 8AVG	*MmElp*123–tRNA EMD-15625	*Mm*Elongator EMD-15626
**Data collection and processing**						
Magnification	105 000×	81 000×	165 000×	105 000×	105 000×	105 000×
Voltage (keV)	300	300	300	300	300	300
Electron exposure (e-/Å^2^)	40	40	42.45	40	40	40
Defocus range	−0.9 to −3.0	−0.9 to −3.0	−0.8 to −2.0	−0.9 to −3.0	−0.9 to −1.5	−0.9 to −3.0
Pixel size (Å)	0.86	1.1	0.81	0.86	0.86	0.86
Symmetry imposed	C1	C1	C1	C1	C1	C1
Initial particle images (no.)	188 389	364 424	693 585	364 424	161 871	581 666
Final particle images (no.)	12 514	128 593	16 809	42 894	24 829	27 128
Map resolution (Å)	4.35	3.7	3.96	4.01	4.35	5.9
FSC threshold	0.143	0.143	0.143	0.143	0.143	0.143
Map resolution range (Å)	3.9 to >10	3.6 to 6.6	3.7 to 9.7	3.8 to >10	2.8 to >10	4.4 to >10
**Refinement**						
Initial model used	6QK7 and 4A8J	4A8J	6QK7	6QK7		
Model resolution (Å)	3.5	3.7	3.5	3.5		
FSC threshold	0.143	0.143	0.143	0.143		
Model resolution range (Å)	n/a	n/a	n/a	n/a		
Map sharpening B factor (Å^2^)	−141.69	−129.16	−67.83	−131.78		
Model composition						
Non-hydrogen atoms	35 795	12 587	24 783	13 749		
Protein residues	4478	1580	2897	1732		
Nucleotide residues			73			
Ligands	1	0	2	2		
*B* factors (Å^2^)						
Protein	99.05	107.76	97.38	84.42		
Nucleotide			254.23			
Ligands	60.85		61.57	55.04		
R.m.s. deviations						
Bond lengths (Å)	0.004	0.004	0.006	0.004		
Bond angles (°)	0.990	0.974	1.014	0.989		
Validation						
MolProbity score	2.13	1.92	2.35	2.17		
Clashscore	12.59	9.75	19.37	13.21		
Poor rotamers (%)	0.02	0.00	0.71	0.07		
Ramachandran						
Favored (%)	90.93	93.80	89.25	90.48		
Allowed (%)	9.00	5.88	10.51	9.34		
Disallowed (%)	0.07	0.32	0.24	0.18		
CC volume	0.65	0.85	0.76	0.71		

### Elongator reconstitution and pull-down assays

Mouse or human Elp123 were incubated with the respective Elp456 subcomplex for 30 min at 25°C with 1:2 weight ratio and subsequent purification on a Superose 6 Increase 10/300 GL column (GE Healthcare) equilibrated with 20 mM HEPES (pH 7.5), 100 mM NaCl and 5 mM DTT. For the pull-down experiments 10 μg of bait and 20 μg of prey were used. Elp123 was immobilized on StrepTactin™ Sepharose™ (Cytiva) beads via Twin-Strep-tagged Elp3 or in case of Elp456 via anti-DYKDDDDK (Pierce™) resin and FLAG-tagged Elp6. The respective subcomplex was added and incubated for 30–60 min at 4°C, followed by three wash steps with 20 mM HEPES (pH 7.5), 100 mM NaCl, 1 mM DTT, 0.05% v/v Tween 20. Elution from StrepTactin or anti-DYKDDDK resin was carried out in the same buffer containing either 5 mM *d*-desthiobiotin or 3× FLAG peptide at a concentration of 300 ng/μl. Input controls and pull-downs were analyzed on a SDS-PAGE and visualized with Coomassie or silver staining. ATP and bulk tRNA were used at indicated concentrations.

### Microscale thermophoresis (MST) and gel shift for tRNA binding assay

The internally Cy5-labeled tRNA Arg^UCU^ (0.2 μM) or tRNA Ala^UGC^ (0.3 μM) were incubated for 5 min at 37°C with serial dilutions of Elp123, Elp456 or Elongator variants (starting from 10 μM) in MST Buffer (20 mM HEPES (pH 7.5), 100 mM NaCl, 5 mM DTT, 0.0125% v/v Tween 20). Measurements were performed at 60% excitation power in Premium Coated capillaries on the Monolith NT.115 (Nanotemper Technologies) at 37°C. Obtained data were analyzed and dissociation constant values were calculated using MO. Affinity software (Nanotemper Technologies) from at least three independent experiments (*n* > 3). For gel shift experiments internally Cy5-labled tRNA Ala^UGC^ (0.2 μM) was incubated with 0.05–1 μM wild type Elp123, Elongator or Elp123_Elp1Δ1132–1224_ variant at 4°C for 15 min. Next samples were separated on NativePAGE™ 4–16% Bis–Tris Protein Gels at 4°C for 90 min and 150 V. Gels were imaged on BIO-RAD ChemiDoc™ MP Imaging System in Cy5 detection mode.

### Acetyl-CoA hydrolysis assay

The assay was performed as previously described (31). In detail, 0.3 μM of Elongator variants were mixed with 2 μM human bulk tRNA in presence of 100 μM acetyl-CoA (ACO) in 1× acetyl-CoA assay buffer and incubated in a thermocycler for 3 h at 37°C. Next, the samples were filtered by a 3 kDa cut-off concentrator (EMD Millipore) and the collected flow-through, of each reaction, was distributed into a 96-well plate. ACO quantity of each sample was determined using the Acetyl-CoA Assay Kit (MAK039, Merck) according to the manufacturer's instructions. Fluorescence intensity was measured on a plate reader (TECAN) at probe-specific excitation (535 nm) and emission (587 nm) wavelengths. Hydrolysis rates were calculated from at least three independent experiments.

### Yeast genetic manipulations and phenotypic characterization

PCR-based deletions of *ELP1*, *ELP3*, *ELP4*, *ELP5* and *ELP6* genes were generated in the UMY2893 wild type strain (2) using a *KlURA3* marker (67) for later counterselection (68). In order to generate the individual mutations in *ELP1*, *ELP3*, *ELP4*, *ELP5* and *ELP6*, each gene was amplified using a primer pair that binds 200 bp up- and downstream of the ORF from UMY2893 strain and ligated into the pJET1.2 blunt vector. Mutations were introduced via site-directed mutagenesis and further verified by DNA sequencing. Subsequently, a *KlTRP1* marker cassette was integrated in the 3′UTR of all mutants constructs using homologous recombination (69). Mutant alleles were reintroduced into the corresponding knockout strains and transformants were selected for the loss of *KlURA3* marker gene by 5-fluoroorotic acid resistance and tryptophan prototrophy. Mutants were further confirmed by PCR and verified by DNA sequencing. To analyze Elongator function in the generated mutant alleles, growth behavior on media was analyzed as previously described (70). Introduction of *ELP1-(c-myc)_3_* and *ELP5-(FLAG)_3_* into the genome was facilitated by homologous recombination of the desired tag together with a *KlLEU2* (67) or clonNAT marker cassette (71). Descriptions and genotypes of all yeast strains are listed in Supplementary Table S1.

### GST-γ-toxin purification

The GST-γ-toxin construct was expressed in the *Escherichia coli* BL21 (DE3) pRARE strain. Cultures were grown at 37°C in autoinduction media (72) and were shifted after 5–6 h to 18°C for overnight induction. Cells were lysed in buffer containing 50 mM Tris–HCl (pH 7.5) at 4°C, 300 mM NaCl, 2 mM DTT and 1 mg/ml lysozyme. After sonication, the lysate was cleared at 40 000 × g for 30 min and the supernatant was cycled on a Protino^®^ 5ml GST/4B column overnight. The column was washed with lysis buffer omitting lysozyme and eluted in the same buffer containing 20 mM glutathione. Eluted fractions were further purified on a Superdex 75 16/600 (GE Healthcare) column, concentrated, and dialyzed into a storage buffer (50 mM Tris–HCl (pH 7.5) at 4°C, 150 mM NaCl, 2 mM DTT).

### *In vitro* tRNA cleavage by γ-toxin

To probe for the mcm^5^s^2^U_34_ modification status of Elongator mutants an *in vitro* cleavage assay using GST-γ-toxin was applied as previously described (73). In detail, tRNA levels were adjusted to the same concentration in cleavage buffer containing 20 mM Tris–HCl (pH 8.0) at 4°C, 150 mM NaCl, 2 mM EDTA and 2 mM DTT. Purified GST-γ-toxin or GST was added to the tRNAs at a final concentration of 4 μM and incubated for 30 min at 30°C. Next, samples were denatured in loading buffer 20 mM Tris–HCl (pH 8.0) at 4°C, 4 M urea, 20 mM boric acid, 2 mM EDTA, 0.02% w/v xylencyanol ff, 0.01% w/v bromophenol blue at 90°C for 5 min and resolved on an 10% urea-PAGE (100 mM Tris–HCl (pH 8.0) at 4°C, 100 mM boric acid, 1 mM EDTA, 10% acrylamide w/v (acrylamide/bis-acrylamide 19:1), 5.12 M urea) at 180 V for 35 min in TBE buffer (100 mM Tris–HCl (pH 8.0) at 4°C, 100 mM boric acid, 2 mM EDTA). tRNA and cleavage products were stained with SYBR™ Gold (Invitrogen).

### Yeast protein extraction, co-immunoprecipitation and western blot analysis

To probe for *in vivo* acetyl-CoA carboxylase (Acc1) interaction, co-immunoprecipitation was carried out as described before (70). 8 mg lysate was incubated with 8 μg antibody coupled Dynabeads (Invitrogen, USA) or anti-FLAG M2 magnetic beads (Sigma-Aldrich, USA) over night. Unbound protein was removed via a magnetic rack and precipitated proteins were washed three times with 1 ml B60 buffer (50 mM HEPES–KOH pH 7.3, 60 mM KAc, 5 mM Mg(Ac)_2_, 0.1% v/v TritonX-100, 10% v/v glycerol, 1 mM NaF, 20 mM ß-glycerolphosphate, 1 mM DTT, cOmplete™ protease inhibitor cocktail (Roche, Germany)). Bound protein was eluted with elution buffer (50 mM Tris pH 8.0, 0.2% w/v SDS, 0.1% v/v Tween 20) at 50°C for 15 min and subsequent incubation of 1× Laemmli buffer at 98°C for 5 min. Proteins were separated on a 10% SDS-PAGE and analysed by Western blotting on a 0.45 μM pore sized polyvinylidene difluoride (Merck, Germany) membrane. Proteins were detected using anti-c-myc (9E10 hybridoma supernatant kindly provided by Prof. Dr M. Maniak), anti-GFP antibody (sc-9996 Santa Cruz, USA), anti-FLAG antibody (F7425 Sigma-Aldrich, USA) and anti-Cdc19 serum (kindly provided by Dr. J. Thorner, University of California, Berkley, USA).

### Sample photo-crosslinking for mass spectrometry

85 μg of *Sc*Elongator complex at 2.22 μM concentration was crosslinked with sulfo-SDA (sulfosuccinimidyl 4,4′-azipentanoate, Thermo Scientific) at 1:500 and 1:1000 sample:crosslinker molar ratios. The reaction was incubated at room temperature for 60 min irradiated with UV light at 365 nm using a Luxigen34 LZ1 LED emitter (Osram Sylvania Inc.) for 10 s, and quenched with a final concentration of 50 mM ammonium bicarbonate (ABC). The crosslinked complex was separated from single subunits on a Novex Bis–Tris 4–12% SDS-PAGE gel (Life Technologies). Gel pieces containing the crosslinked complex were excised. The sample was reduced with DTT and free sulfhydryl groups were alkylated using iodoacetamide (74). Proteins were digested overnight at 37°C with 1 μg trypsin (Thermo Scientific Pierce) per 20 μg of protein sample. The digested peptides were recovered and desalted using C18 StageTips (75). The eluates of the StageTips were pooled and fractionated by size exclusion chromatography (SEC) using a Superdex™ 30 Increase 3.2/300 column (GE Healthcare) and a mobile phase consisting of 30% v/v ACN and 0.1% v/v trifluoric acid at a flow rate of 10 μl/min. The first six fractions (50 μl each) containing peptides were collected and the solvent was removed using a vacuum concentrator.

### Crosslinking mass spectrometry data acquisition

For the liquid chromatography–tandem mass spectrometry (LC–MS/MS) analysis, an Ultimate 3000 RSLCnano system (Dionex, Thermo Fisher Scientific) connected to an Orbitrap Fusion Lumos Tribrid mass spectrometer (Thermo Fisher Scientific) was used. Each SEC fraction was resuspended in 1.6% v/v ACN and 0.1% v/v formic acid before injection onto a 50-cm EASY-Spray C18 LC column (Thermo Scientific, 50°C operating temperature). The flow rate during sample loading and separation was 0.3 μl/min. The mobile phase consisted of solvent A (0.1% v/v formic acid) and B (80% v/v ACN, 0.1% v/v formic acid). A 2–55% gradient of B was applied over the course of 90–100 min (optimised per SEC fraction) with a final increase to 95% B within 2.5 min. An EASY-Spray source (Thermo Scientific) ionised the eluting peptides before introducing them to the mass spectrometer. For each SEC fraction, two acquisitions were carried out. Data-dependent MS data were acquired in cycles of 2.5 s using the top-speed setting and the full scan mass spectrum was recorded in the Orbitrap with a resolution of 120 000. Ions with a charge state from 3+ to 7+ were selected for fragmentation by stepped higher-energy collisional dissociation (26%, 28% and 30%) using a decision tree (76). Spectra of fragments were recorded in the Orbitrap with a resolution of 60 000. Peaks were excluded after a single repeat for the duration of 60 s (dynamic exclusion).

### Crosslinking mass spectrometry data processing

MS2 peak lists were generated from the mass spectrometric raw data with the MSConvert module of ProteoWizard (version 3.0.11729). Precursor and fragment m/z values were recalibrated based the average mass error of linear peptide spectrum matches. Additional peptide modifications were identified using FragPipe (version 18.0) with MSFragger (version 3.4; (77)) and philosopher (version 4.3.0; (78,79). The search was performed with the ‘Open’ pre-set workflow but with Crystal-C disabled. Crosslinked peptides were identified using xiSEARCH (version 1.7.6.4; (80)) using a database comprising 100 proteins with the highest intensity-based absolute quantification identified in the same experiments by MaxQuant (version 2.0.3.0; (81)). The search was performed using the following parameters: Enyzme: Trypsin; Missed Cleavages: 3; Missing Mono-Isotopic Peaks: 2; Cross-Linker ¦ Mass: NonCovalent ¦ 0, SDA_Knterm ¦ 82; MS Tolerance: 3 ppm; MS2 Tolerance: 5 ppm; Fixed Modifications: carbamidomethylation (C); Variable Modifications: SDA-hydro (K, S, T, Y), SDA-loop (K, S, T, Y), acetylation (K, C, S, T, N-terminus), acetaldehyde + 26 (H, K, N-terminus), carboxyamidomethylation (M, C, U), deamidation (N, R, Q), amidination (K, N-terminus). Search results were filtered using xiFDR (version 2.1.5.6; (82,83)). Prior to FDR calculation, prefilters were applied on both target and decoy matches (peptide1 unique matched conservative > 2.0; peptide2 unique matched conservative > 2.0; peptide1 unique crosslinked matched non lossy > 0.0; peptide2 unique crosslinked matched non lossy > 0.0). The results were then filtered to a false discovery rate (FDR) of 3% on the residue-pair level, resulting in 1047 residue pairs (119 heteromeric). FDR settings at lower error levels were optimised by enabling boosting for heteromeric crosslinks.

## RESULTS

### Single particle cryo-EM structure of yeast Elongator

**Figure 1.**
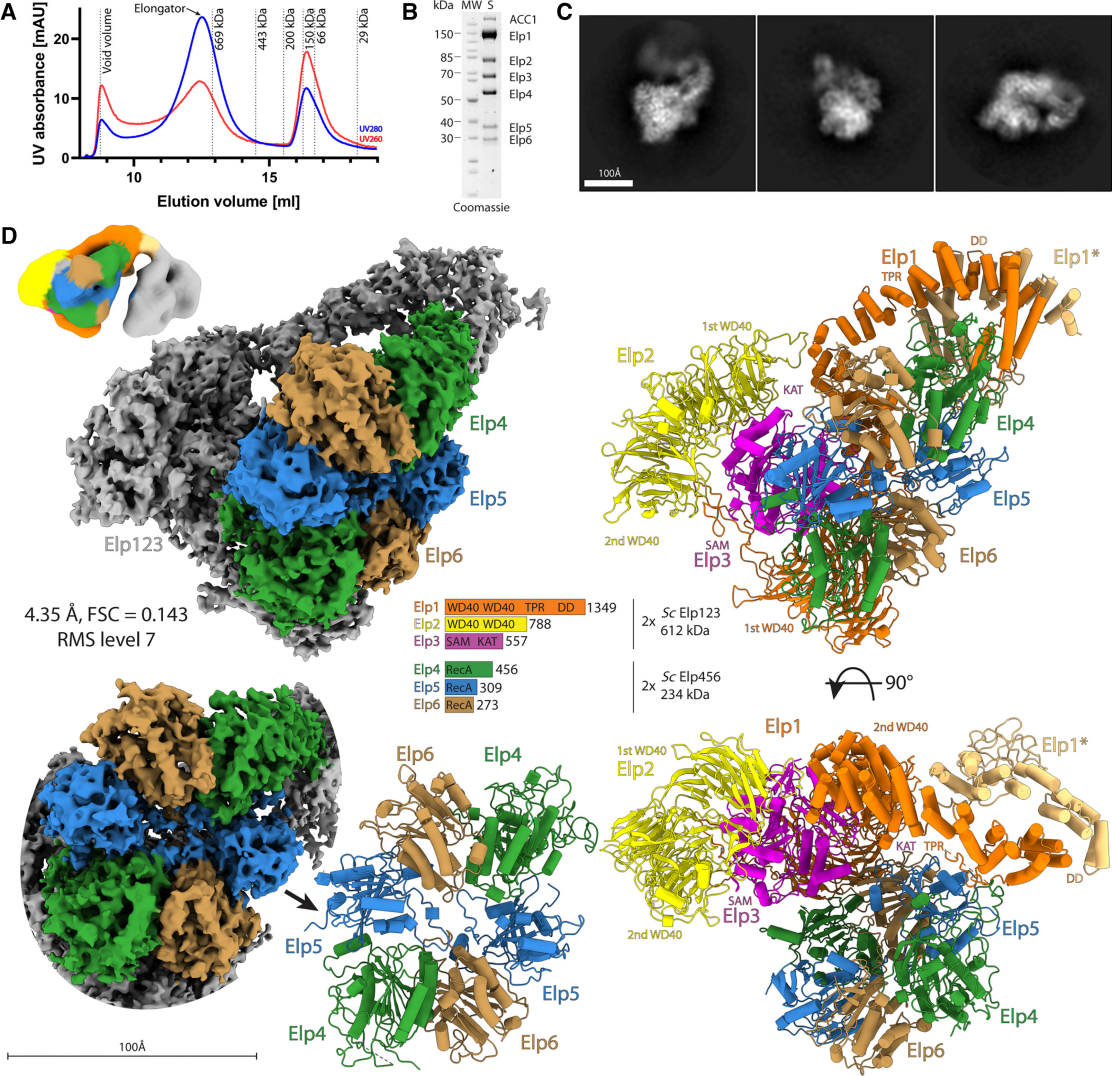
Cryo-EM structure of yeast Elongator. (**A**) Purification chromatogram profile of yeast Elongator. Red and blue lines represent UV260 and UV280 absorbance, respectively. The peak corresponding to Elongator is indicated with a black arrow. (**B**) SDS-PAGE representing the final purification quality of the Elongator sample. (**C**) Reference-free 2D class averages of yeast Elongator data set, scale bar 100 Å. (**D**) Cryo-EM reconstruction of yeast Elongator at 4.35 Å resolution visualized at indicated map contouring with the atomic model at two orientations (upper and lower right). A schematic representation of the complex is shown in the middle with Elp456 highlighted in color on the cryo-EM reconstruction and all subunits colored in accordance with the scheme on the atomic model. Front view on the density and atomic model of Elp456 (lower left). Double lobe low-resolution cryo-EM reconstruction of Elongator (upper left), coloring corresponds to the atomic model of native *Sc*Elongator.

The endogenous Elongator complex is constitutively expressed in yeast cells, but its low abundance has previously complicated the purification of the stoichiometric complex in quantities required for comprehensive structural and biochemical analyses (37,84). To overcome this technical limitation, we adapted an advanced overexpression system for *S. cerevisiae* Elongator (*Sc*Elongator) enabling us to simultaneously overexpress the full-length versions of Elp1, Elp2, Elp3, Elp4, Elp5 and Elp6 in suitable yeast strains (Supplementary Figure S1A). The incorporation of specific affinity tags for Elp1, Elp3 and Elp6 allowed us to establish a robust purification protocol that reproducibly resulted in pure and homogenous samples of fully assembled *Sc*Elongator containing all six subunits (Figure 1A, B and Supplementary Figure S1B). We typically obtained 5–15 μg of purified complex per liter of yeast culture, which was sufficient for biochemical and structural analyses by single particle cryo-EM. The purified stoichiometric complex eluted from a size exclusion chromatography column at a calculated molecular weight of ∼850 kDa. Like the endogenous complex (85), the recombinant Elongator contains two copies of each subunit. The 260/280 nm absorption ratio indicates that the purified complex had no co-purified endogenous tRNAs nor other nucleic acids (Figure 1A). We observed a higher molecular weight protein at ∼250 kDa, which we identified as Acetyl-CoA carboxylase 1 (Acc1) by mass spectrometry. Acc1 is a multifunctional homodimeric enzyme of approximately 500 kDa that catalyses the formation of malonyl-coenzyme A from ACO (86). To check whether yeast Acc1 interacts with the Elongator *in vivo*, we tagged endogenous Elp1 (c-myc), Elp5 (FLAG) in an Acc1 (GFP) background (87) and conducted co-immunoprecipitation (co-IP) assays. As we did not observe any interaction of Acc1 with Elongator in these assays (Supplementary Figure S1C), we conclude that Acc1 is an co-purifying contaminant in our preparations. Of note, we also co-purified sub-stoichiometric amounts of Kti11 and Kti12, whereas other regulatory proteins known to temporarily associate with yeast Elongator were undetectable (88). Furthermore, we did not find any hits for actin, tubulin, RNA polymerase subunits or histones, which have been proposed as potential Elongator binding partners in other studies (29,89–91).

Next, we prepared cryo-EM grids with the freshly purified sample and analyzed the structure of the complex using single particle cryo-EM. After vitrification and grid optimization, we collected a dataset of 6339 micrographs on a Titan Krios microscope. We analyzed the complete dataset by applying basic image curation steps as well as iterative rounds of particle picking, 2D/3D classification routines and refinement procedures. Thereby, we determined the structure of *Sc*Elongator at an overall resolution of 4.35 Å (Figure 1C, D, Supplementary Figure S2A and Table 1). The reconstruction shows an additional, unambiguous density for the *Sc*Elp456 ring next to one of the two Elp123 lobes. In an attempt to further improve the resolution of the reconstruction, we stabilized the sample with a low concentration of BS3. However, the stabilized sample resulted in a reconstruction at a lower overall resolution of 6.6 Å. The maps from both approaches show a very similar overall shape of the complex, independently confirming the position of Elp456 after two different routes of sample preparation (Supplementary Figure S2B). The dimensions of the complex are ∼200 × 162 × 140 Å, in line with the estimation from a previous low-resolution negative stain reconstruction (27). The local resolution estimation indicates that the center of the Elp123 lobe extends to 3.9 Å, whereas parts of Elp456 as well as the TPR and dimerization region of Elp1 are less well resolved. Similar to our previous high-resolution reconstructions of yeast Elp123 (with and without bound tRNA), the density of the second Elp123 lobe is not clearly visible. In addition, 2D class averages already show that the second Elp123 lobe is blurred after image averaging, suggesting relatively high flexibility between the two Elp123 lobes, which is also observable in presence of Elp456. Nevertheless, we managed to reconstruct a map for the complete complex with both lobes visible at an estimated overall resolution of 17.2 Å, when stabilized with BS3 (Figure 1D). As we could only visualize the second lobe at low map thresholds and did not see any density of Elp456 in the second lobe, we decided to exclude the empty lobe from subsequent refinement procedures. In summary, our results reveal the overall arrangement of the yeast Elongator complex at an unprecedented resolution. Furthermore, the cryo-EM structure(s) independently confirm that the hexameric ring-shaped Elp456 subcomplex is indeed asymmetrically localized on only one lobe. The high quality of our cryo-EM map shows details of the complex and allows us to unambiguously determine the orientation of the *Sc*Elp456 subcomplex. Additionally, the presented maps allow for the precise structural characterization of all individual Elongator subunits, their relative position and their interaction network within the fully assembled complex at high resolution (Figure 1D).

### Elp456 bridges between Elp3 and the C-terminus of Elp1

**Figure 2.**
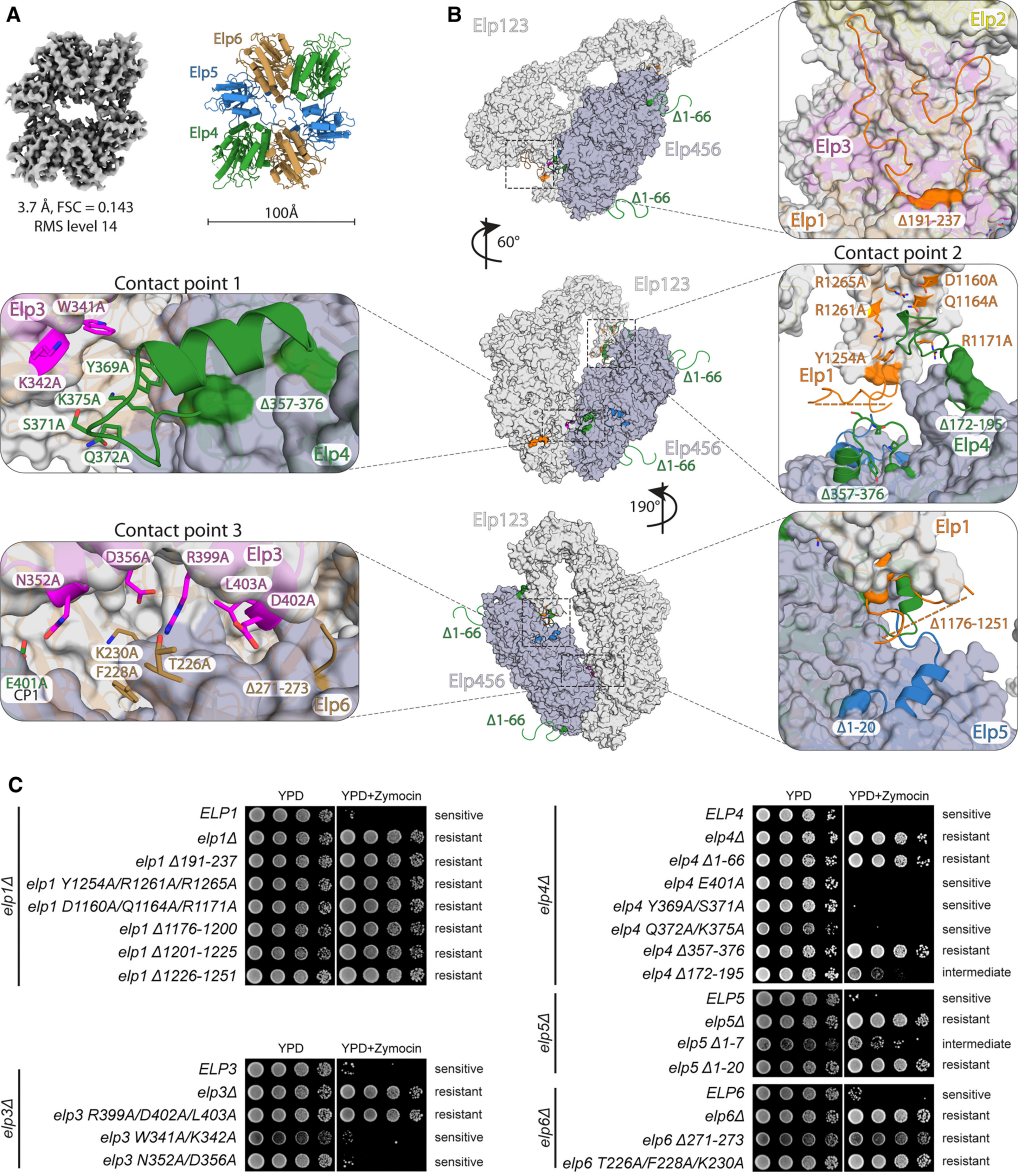
Functional validation of yeast Elongator. (**A**) Cryo-EM map and atomic model of the apo *Sc*Elp456 subcomplex. (**B**) Front view on the atomic model of yeast Elongator (left). Atomic model in surface representation at different orientations with a position of selected mutations represented in sticks (point mutation) and cartoon (deletion or truncation). Colouring corresponds to the affiliation of the particular position to the given subunit. (**C**) Phenotypic analyses of various yeast strains carrying variations in Elp1, Elp3, Elp4, Elp5 and Elp6 using zymocin resistance assays.

The cryo-EM structure of yeast Elp456 in complex with Elp123 almost perfectly resembles the previously solved structures of the truncated Elp456 hexamer determined by macromolecular crystallography (85,90). As we observed unbound ring-shaped Elp456 particles in one of our datasets, we also determined the cryo-EM structure of free full-length *Sc*Elp456 at 3.7 Å overall resolution (Figure 2A, Supplementary Figure S3A and Table 1). The newly obtained structures of full-length Elp456 are almost identical to the truncated crystal structure of the Elp456 subcomplex (Supplementary Figure S3B). The structure confirms that Elp456 contains two copies of Elp4, Elp5 and Elp6, forming a symmetric dimer of trimers. However, the N- and C-terminal extensions of Elp4 and Elp5 (including a small predicted domain at the C-terminus of Elp5 (85)) remain flexible also in the fully assembled complex. In addition, we compared the structure of the Elp123 subcomplex after binding of Elp456 with the previously determined Elp123 subcomplex in absence and presence of tRNA (31). We did not detect any major rearrangements of the Elp123 subcomplex after binding of Elp456 (Supplementary Figure S3C), although the lobe adopts a slightly less compacted topology. As mentioned above, we did not observe a stabilization of the second Elp123 lobe upon recruitment of Elp456 to the Elp123 dimer. As previously anticipated, the two copies of the largest subunit, namely Elp1 (∼150 kDa), act as the central scaffold for the lobe and the whole Elongator assembly. The two N-terminal WD40 domains of each Elp1 molecule create a platform for the catalytic Elp3 subunit, which is clamped from the opposite side by Elp2. In our map, we observed an additional unaccommodated density in the proximity of the rSAM domain of Elp3, into which we fit a previously uncharacterized loop region of the first WD40 domain of Elp1 (aa191–237). This unanticipated loop stretches from the third β-sheet of the WD40 domain along Elp3 towards the base of Elp2 and returns to the fourth β-sheet of the same WD40 domain (Figure 2B). Characteristic densities for the iron-sulfur cluster are clearly recognizable in both native and crosslinked structures of *Sc*Elongator. However, we were not able to identify densities for bound SAM or 5′-dA molecules, which were present in proximity to the cluster in the previous cryo-EM structure of the Elp123 subcomplex (Figure 1D) (92).

Our Elongator cryo-EM structure reveals that the hetero-hexameric Elp456 occupies a position that faces the active site of Elp3 and bridges between the enzymatic subunit and the C-terminal region of Elp1. Both copies of Elp4 and one copy of Elp5 and Elp6 each are interacting with the Elp123 subcomplex. Although Elp2 does not seem to be directly involved in Elp456 binding, it might contribute indirectly by stabilizing the Elp123 lobe (93). Furthermore, the resolved structure of Elongator is in full agreement with spatial restraints derived from a cross-linking mass spectrometry analysis of TAP-tag purified endogenous Elongator complex (27). In detail, Elp123 and Elp456 subcomplexes interact with each other at three individual contact points involving Elp1, Elp3, Elp4, Elp5, and Elp6. The first contact point (CP1) is formed between less conserved residues of the Elp3 rSAM domain (Trp341 and Lys342) and a loop region in Elp4 (Tyr369, Ser371, Gln372, Lys375 and Glu401). The second contact point (CP2) is formed between a loop region of Elp4 (aa172-195) and helices α27 and α28 of the dimerization domain of Elp1 (Asp1160, Gln1164, Arg1171, Tyr1254, Arg1261 and Arg1265). This larger interface also involves the N-terminus of Elp5 and a not well-defined loop region of Elp1 (aa1176-1251) known to carry functionally relevant phosphorylation sites (35). The third contact point (CP3) is an interface that is created at the three-way junction between the KAT domain of Elp3 and the C-terminal residues of Elp6 (Figure 2B). Noteworthy, the N-terminus of Elp4 (Elp4 1–66), which was shown to contribute to the interaction with Elp1 (27), was not visible in our densities and did not participate in any long-range interactions with Elp1. All interacting residues of Elp456 are involved only once in the interaction with Elp123, and the respective second copy of the residues within the ring appear to be irrelevant for the respective interactions. Therefore, we were able to mutate selected residues and regions to analyze their specific role for the *in vivo* function of Elongator (Figure 2B, C).

### The interaction interfaces between Elp123 and Elp456 are crucial for Elongator's tRNA modification activity

We generated numerous yeast strains that carry specific single amino acid substitutions or deleted regions in Elp1, Elp3, Elp4, Elp5 and Elp6. Next, we tested these strains in previously established phenotypical assays that indirectly probe the tRNA modification activity of Elongator *in vivo* (2,94,95). In addition, we used an *in vitro* γ-toxin tRNA cleavage assay to further validate the observed phenotypes of the structure-guided mutations (73) (Supplementary Figure S4A). γ-toxin (the active tRNase subunit of trimeric killer toxin zymocin) acts as a nuclease that requires the mcm^5^s^2^U_34_ modification to cleave tRNAs at the anticodon. Therefore, any mutation that reduces or abolishes the tRNA modification activity of Elongator, leads to reduced mcm^5^s^2^U_34_ modification levels *in vivo* and protects tRNAs from cleavage by γ-toxin (95,96). Since the readout of the aforementioned γ-toxin-based assays predominantly samples the modification status of tRNA Glu^UUC^, we also monitored the Elongator dependent mcm^5^U_34_ modification of the *SUP4* nonsense suppressor tRNA Tyr^UUA^ via its efficiency of ochre stop codon readthrough (2). As expected, gene deletions of *elp1*, *elp3*, *elp4*, *elp5*, and *elp6* were found to be resistant to zymocin and the toxic arginine analogue canavanine in a *SUP4* tRNA and *can1-100* allele containing background (for more details, see (2)), Figure 2C and Supplementary Figure S4B, C). Furthermore, we show that the newly identified loop (aa191–237) from the first WD40 of Elp1 and the unstructured N-terminal region of Elp4 (aa1–66) are crucial for Elongator function *in vivo*.

Next, we analyzed the structure-guided mutation in the three identified contact points CP1, CP2 and CP3 to understand the importance of these regions individually. The mutations of Elp3 residues ( *W341A/K342A)* and Elp4 (*E401A*, *Y369A/S371A* and *Q372A/K375A*) in CP1 show no measurable effects on the tRNA modification activity of Elongator. Strikingly, all tested mutations in CP2 nearly abolished Elongator's tRNA modification activity. Mutations and deletions of the identified residues in Elp1 (*Δ191–237*, *Y1254A/R1261A/R1265A, D1160A/Q1164A/R1171A, Δ1176–1200, Δ1201–1225, Δ1226–1251*), Elp4 (*Δ172–195, Δ357–376)* and Elp5 (*Δ1–20)* display tRNA hypo-modification phenotypes that are indistinguishable from their respective gene deletion mutants, in all conducted *in vivo* and *in vitro* tRNA modification assays (Figure 2C and Supplementary Figure S4). The third and most central interface (CP3) appears to be as important for Elongator activity as CP2. Mutations in Elp3 (*R399A/D402A/L403A)* and Elp6 (*Δ271–273, T226A/F228A/K230A*) are resistant to growth inhibition by zymocin. Of note, mutants in Elp123 demonstrate identical phenotypes in all applied assays, whereas individual mutations in Elp456 (e.g. *elp4 Δ172–195*, *elp5 Δ1–7*, *elp6 Δ271–273* and *elp6 T226A/F228A/K230A)* exhibited a slightly divergent phenotype. These variations mostly affect mutants that display intermediate phenotypes and might occur due to different sensitivity of performed assay formats. These results also support the recent observation that Elp456 function might only be relevant for a subset of cellular tRNAs (22). All zymocin and canavanine-sensitive mutants and the latter mentioned variable phenotype mutants are susceptible to *in vivo* and *in vitro* γ-toxin cleavage. In conclusion, our data show that interfaces of CP2 and CP3 are crucial for Elongator's tRNA modification activity, whereas the CP1 region, between Elp3 and Elp4 appears to be functionally dispensable. These results directly validate our structural model that depicts the complicated interaction network between individual subunits of the assembled Elongator complex.

To further validate our structural model we carried out a photo-crosslinking mass spectrometry (XL-MS) analysis of the purified yeast Elongator complex. 638 out of 883 detected cross links are in agreement (30 Å distance) with our structural model (Supplementary Figure S5A). In addition, 125 out of 189 crosslinks that disagree with our model are located in loop regions of Elp1, namely aa191–237 and aa1176–1251. Therefore, it seems these loops can become flexible, which is in agreement with the previous high-resolution reconstruction of yeast Elp123 (92) where no density was detectable for them. We also checked for any specific crosslinks between Elongator and Acc1, but only a single crosslink was detected, indicating that the two complexes indeed do not interact or share a large interface. Although, the second Elp123 lobe is not well-resolved in our maps the presence of a few crosslinks indicated that the two Elp123 lobes could get in close proximity of each other also in the presence of Elp456. Hence, we used additional 3D variability and masked 3D classification analyses to check whether the second lobe can obtain any specific conformations upon Elp456 binding (Supplementary Figure S5B). We indeed managed to partially enhance the density of the second lobe, but due to reduced number of particles the resolution decreased (Supplementary Figure S5B). None of the obtained reconstructions revealed a complete second lobe or specific conformation, which is consistent with the low resolution of the two-lobed BS3 stabilized Elongator complex (Figure 1D). These additional results further support our notion that the second lobe stays flexible in the fully assembled Elongator complex.

### The structure of the Elp123 subcomplex is highly conserved among eukaryotes

Like in yeast, the murine Elongator consists of six subunits. However, the sequence similarity and identity of the mouse proteins vary between the subunits compared to their yeast counterparts. Although Elp1, Elp2, Elp3 and Elp4 proteins show high sequence identity, Elp5 and Elp6 display surprisingly low sequence similarity among eukaryotes (84). Therefore, we asked whether the Elongator complex is structurally and functionally conserved in higher eukaryotes, particularly in mammals. We employed the biGBac expression system to overproduce mouse subcomplexes by co-expressing *Mm*Elp123 and *Mm*Elp456 genes separately in insect cells. The incorporation of affinity tags on Elp3 (TST) and Elp6 (FLAG) facilitated the purification of the individual subcomplexes using standard affinity and size exclusion chromatography. The gel filtration profiles of *Mm*Elp123 (∼610 kDa) and *Mm*Elp456 (∼220 kDa) show that they all elute at the expected elution volume (Supplementary Figure S6) demonstrating that the mouse complex also form dimers like the yeast counterparts.

**Figure 3.**
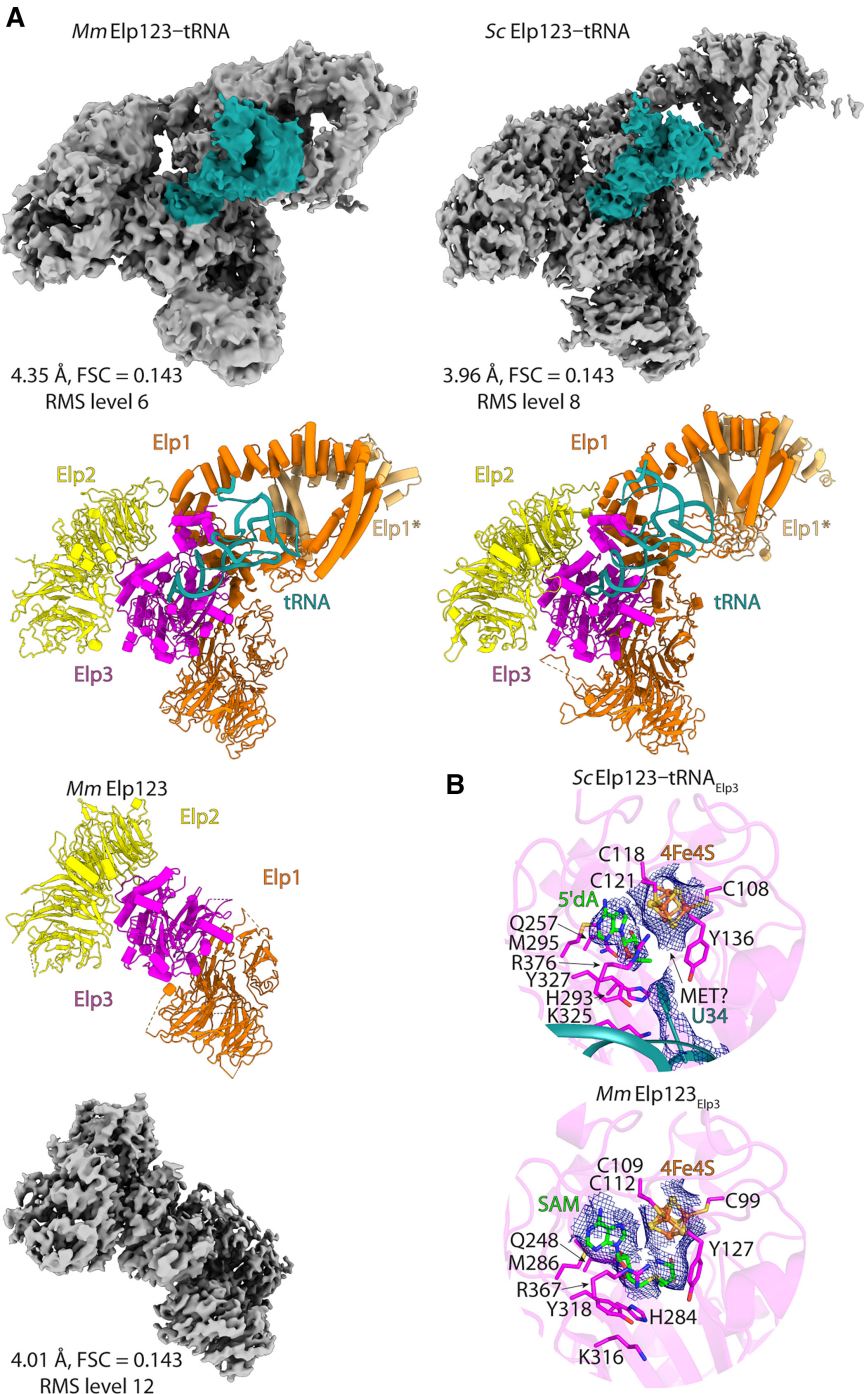
Elongator structural conservation in eukaryotes. (**A**) Cryo-EM reconstructions of *Mm*Elp123–tRNA (upper left), *Mm*Elp123 (lower left) and *Sc*Elp123–tRNA (upper right) complexes with atomic models in the same orientation. tRNA colored in deep teal. (**B**) Comparison of the active site of *Sc*Elp123–tRNA and *Mm*Elp123. Selected, structurally conserved residues in stick representation, with densities shown for iron-sulfur clusters (*Sc*Elp123–tRNA/*Mm*Elp123), 5′-dA (*Sc*Elp123–tRNA) and SAM (*Mm*Elp123).

To determine the structure of *Mm*Elp123, we vitrified freshly purified samples in absence and presence of *in vitro* transcribed mouse tRNA Arg^UCU^. We optimized the sample preparation procedure, collected large datasets and solved structures that are with and without tRNA at 4.35 Å and 4.0 Å resolutions, respectively (Supplementary Figure S7A,B and Table 1). Meanwhile, we also collected a dataset for *Sc*Elp123 in complex with yeast tRNA Ala^UGC^ and obtained a reconstruction at an improved resolution of 3.96 Å (Supplementary Figure S8 and Table 1). This new tRNA-bound structure of *Sc*Elp123 allowed us to build a more reliable atomic model of the tRNA-bound complex (Figure 3A), which facilitated the structural comparison of individual regions in *Sc*Elp123 and *Mm*Elp123. Both, yeast and mouse Elp123–tRNA structures, contain characteristic L-shaped tRNA densities, spanning from the center of Elp3 to the C-terminus of Elp1 (Figure 3A and Supplementary Figure S9). tRNA molecules are positioned in both complexes in a similar conformation – the ASL is inserted in the active site of Elp3 and the T-arm is in contact with the CTD of Elp1, while the acceptor stem points away from the complex. The second Elp123 lobe remains flexible and poorly defined, in both structures. Thus, tRNA binding does not affect the relative orientation between the two lobes, and the second lobe was excluded from the subsequent refinement process. Of note, we used masked 3D classification to identify a subfraction of particles that shows a tRNA-like density also in the second Elp123 lobe. Nonetheless, due to necessary subclassification procedures the resulting maps lack the quality to ultimately confirm that mouse Elp123 can simultaneously bind tRNAs in each of its lobes, like yeast Elp123 (92). As for the mouse apo Elp123 structure, the obtained density is significantly smaller and only the core lobe of the subcomplex is structured, resulting in decreased overall dimensions (∼140 × 100 × 65 Å; Figure 3A). In detail, the spatial arrangement of Elp2, Elp3 and the N-terminal region of Elp1 are very similar to the tRNA-bound *Mm*Elp123 and the *Sc*Elp123 structures (92). Strikingly, the CTD of *Mm*Elp1, which is visible in the yeast Elp123, remains flexible in *Mm*Elp123 in the absence of tRNA (Figure 3A). This observation indicates that yeast Elp123 is inherently more stable in the absence of tRNAs and that the binding of tRNAs induces structural rearrangements in the Elp1 CTD of mammals. Nonetheless, both yeast and murine Elp123 appear very similar in its tRNA bound form. Our comparative analyses of the tRNA-bound *Sc*Elp123 and *Mm*Elp123 show that both subcomplexes bind their tRNA substrates in an almost identical fashion and that the involvement of several regions in tRNA binding might have contributed to the high sequence conservation in Elp1 and Elp3 amongst eukaryotes.

As aforementioned, the enzymatically active Elp3 subunit shows a high overall sequence conservation. This similarity becomes even more evident, by comparing the structural details of the active sites of *Sc*Elp123 and *Mm*Elp123 (Figure 3B). Both complexes harbor an iron-sulfur-cluster [4Fe4S] involved in the generation of a 5′-dA radical, which is necessary for the U_34_ modification reaction, and the cluster is coordinated by three highly conserved cysteine residues, which contact the respective iron atoms of the cluster. Although we did not supplement the sample with SAM before vitrification, we identified a bound 5′-dA molecule in the yeast active site next to the cluster. Gln257 contacts one of the hydroxyl groups in 5′-dA and positions the radical carrying methyl group in close proximity to Tyr136 and Tyr327, which both are positioned in a similar distance to the base of U_34_ and to the methyl group of 5′-dA. Furthermore, we observed that the density of Tyr136 connects to the one of U_34_, suggesting direct interaction. This tyrosine possibly pulls the uridine closer to the iron-sulfur cluster and the 5′-dA molecule. As for the fourth iron atom of the cluster, it is not coordinated by any cysteine residue but it is bordered by an additional, adjacent density, which most likely can be attributed to bound methionine. Methionine is the second cleavage product of SAM which was also reported in crystal structures of the bacterial rSAM proteins RlmN (97) and MiaB (98). Surprisingly, we could identify an intact SAM molecule bound to the iron-sulfur cluster of *Mm*Elp123, which was co-purified from the host cells. The coordination of this ligand is similar to the 5′-dA molecule found in *Sc*Elp123. At the current stage, it remains unclear if the difference results from different catalytic rates, applied classification schemes or from the fact that we isolate *Sc*Elp123 from yeast cells, whereas *Mm*Elp123 was produced in insect cells, though in case of archaeal Elp3 SAM remained uncleaved in the absence of tRNA (34). Nonetheless, the structural similarities between the subcomplexes (pre and post SAM cleavage), suggest that the cleavage reaction itself does not induce major rearrangements in the active site of Elp3 or in the coordination of the adenosine moiety. Thus, our detailed analyses of the active sites in *Sc*Elp3 and *Mm*Elp3 further highlight the homology of yeast and mouse Elongator complexes.

### The structure of the fully assembled Elongator is highly conserved among eukaryotes

**Figure 4.**
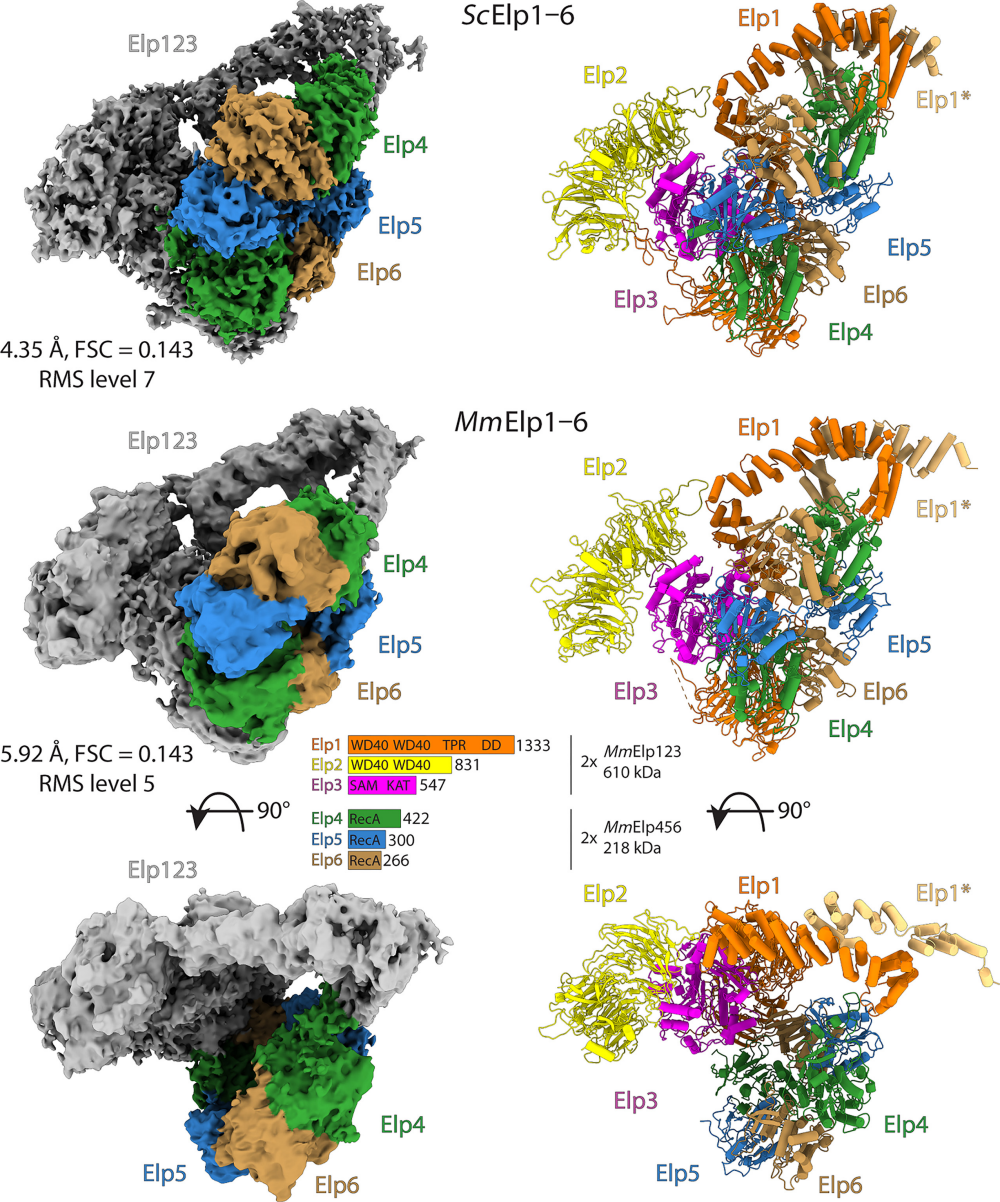
Mouse Elongator complex. Cryo-EM reconstructions of *Mm*Elongator complex with an atomic model in the same orientation. Elp456 is colored in accordance to the scheme from Figure 1D. For comparison, yeast density and model are in the same orientation above.

Finally, we also investigated whether in mouse Elp456 interacts with Elp123 in a similar way as in yeasts. For this, we reconstituted the Elongator *in vitro* from individually purified *Mm*Elp123 and *Mm*Elp456 subcomplexes and analyzed the obtained complex by single particle cryo-EM (Figure 4, Supplementary Figures S10 and S11A). In detail, we observed efficient complex formation after combing both purified subcomplexes without the addition of other co-factors. The reconstituted complex shows a stoichiometric composition of all six subunits, indicating that the bi-lobal Elp123 in mouse Elongator also preferentially binds only one hetero-hexameric Elp456 subcomplex. Subsequently, we used the reconstituted and re-purified complex after gel-filtration to prepare cryo-EM grids. After sample optimization and data collection, we determined the cryo-EM structure of the mouse Elongator at an overall resolution of 5.9 Å (Table 1). The structure of *Mm*Elongator is very similar to yeast Elongator, with a clearly distinguishable lobe, an arch region between the lobes and an asymmetrically positioned Elp456 ring on one of the two Elp123 lobes. The quality of the obtained map allowed the unambiguous placement of the Elp456 subcomplex, revealing the same three contact points formed between the subcomplexes in yeast. However, the limited resolution of our reconstruction prevented us from analyzing the molecular details of the active site in the fully assembled mouse Elongator complex.

**Figure 5.**
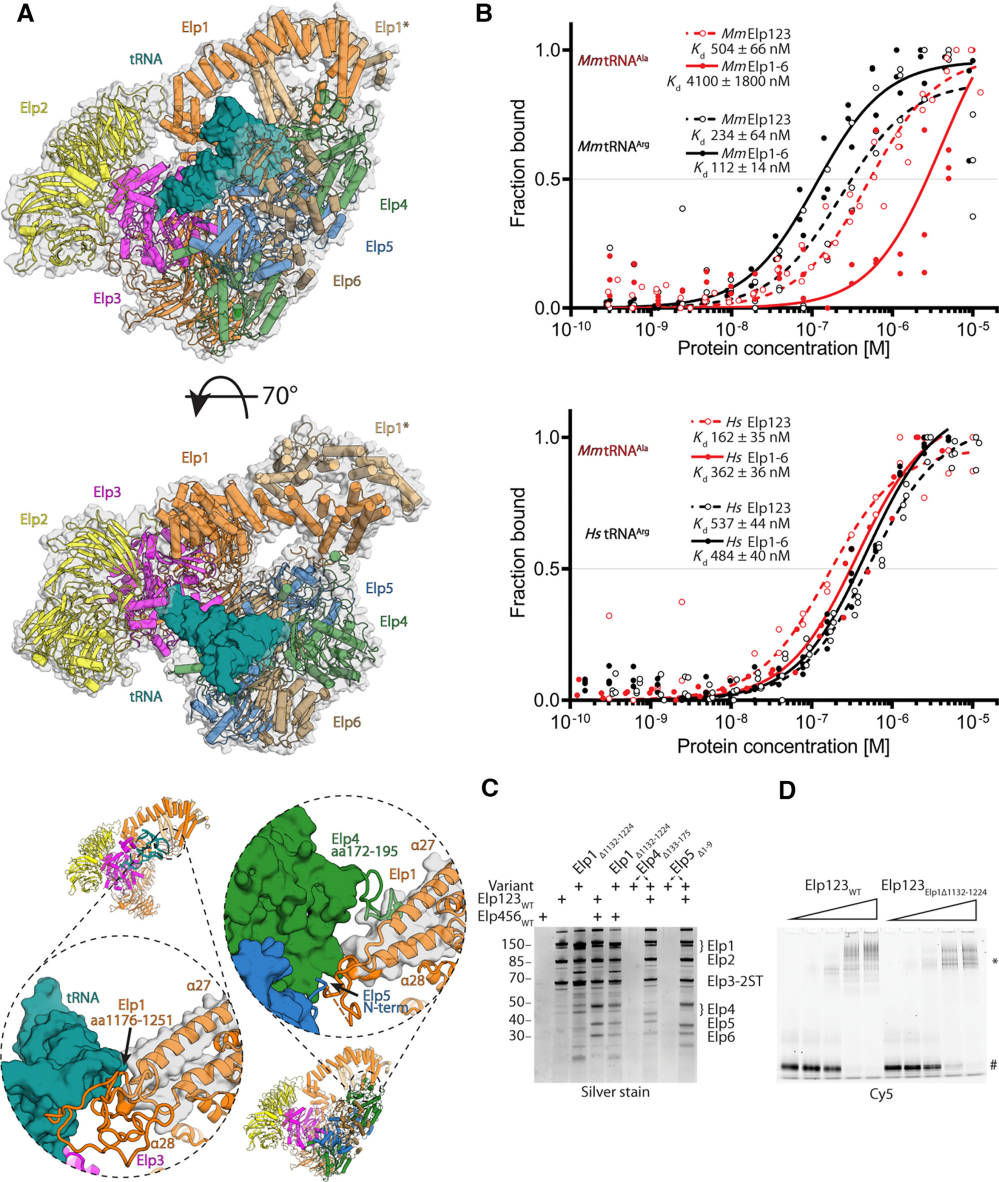
Biochemical and functional characterization of Elongator complex from higher eukaryotes. (**A**) Superposition of *Sc*Elp123–tRNA and *Sc*Elongator in two orientations, showing a possibility of the tRNA (deep teal) binding by the latter complex (above). Below: yeast CP2 comparison between *Sc*Elp123–tRNA and *Sc*Elongator complexes showing Elp1 tRNA binding loop displacement. (**B**) Microscale thermophoresis measurements, respective fits, and calculated dissociation constant (*K*_d_) values for the mouse (top) and human (bottom) Elp123 (red) and Elongator (black). In both cases, the Hill coefficient is close to 1, indicating the presence of independent binding sites. *n* = 3. (**C**) SDS-PAGE gel comparing *in vitro* reconstitution of human Elongator complex with the use of Elp1, Elp4 and Elp5 functional variants of CP2 structural features presented in the previous panel. Twin-Strep-tag indicated on Elp3 subunit. (**D**) EMSA assay using wild type human Elp123 (left), human Elp123_Elp1Δ1132–1224_ (right), and fluorescently labeled tRNA Ala^UGC^. The positions of free tRNA and protein–tRNA complexes (*) are indicated next to the native polyacrylamide gel electrophoresis.

We would like to highlight that although the fully assembled Elongator binds tRNAs (31), despite our intense experimental efforts, we were still not able to attain the yeast or mouse tRNA-bound Elongator structure. To explore whether Elongator could in principle accommodate tRNA molecules in an Elp456-occupied Elp123 lobe, we superimposed *Sc*Elp123–tRNA on *Sc*Elongator via its Elp3 subunit (RMSD 1.22 Å^2^) (Figure 5A). The superimposition showed that the tRNA molecule would fit without any significant clashes into the space formed between the two subcomplexes and that the binding of the ASL in the active site of Elp3 would be unaffected. Moreover, the relatively long and not well-structured loop region (aa169–233) in Elp4, which binds the CTD of Elp1, might provide additional flexibility to accommodate a tRNA molecule. In addition, our *Sc*Elongator model also clearly shows that the tRNA binding loop (aa1176–1251) of Elp1 is displaced by Elp456 and would not be able to interact with the T-arm of tRNAs (Figure 5A). This observation is in line with the fact that *Sc*Elongator has a lower tRNA binding affinity compared to the *Sc*Elp123 subcomplex (31). Of note, the second lobe of Elp123, which is not occupied by Elp456, would in principle be able to bind tRNA, strongly complicating the interpretation of the results from the binding assays.

### The CTD of Elp1 is responsible for tRNA binding and subcomplex interaction

We further investigated the importance of these structural rearrangements by complementary biochemical analyses. We used human Elongator subcomplexes produced in insect cells and reconstituted them as we did with the mouse Elongator sample (Supplementary Figures S6B and S11A). First, we measured the binding affinity of mouse and human Elp123 and Elongator towards *in vitro* transcribed (IVT) tRNAs. Like yeast Elp123 (31), mouse and human Elp123 bound mouse tRNA Ala^UGC^ with higher affinity than the fully assembled Elongator complex, but when we used mouse or human tRNA Arg^UCU^ we observed no differences in affinity (Figure 5B). These data again support the notion that Elp456 might discriminate between sub-pools of tRNAs (22). Second, we examined whether the recombinantly produced mouse and human Elongator assemblies hydrolyze (ACO) faster upon tRNA binding (33). Initially, we employed HEK293 cells-derived human bulk tRNA for the assay, and it did not stimulate the ACO activity of yeast Elongator (Supplementary Figure S11B). To exclude any incompatibility of *Sc*Elongator with human-specific tRNA modification patterns, we then tested yeast samples with extracted yeast bulk tRNA. Our results show that all used Elongator complexes can hydrolyze ACO in the presence of tRNAs to different degree. Elongator displays slightly lower activity than their respective Elp123 counterparts, but a high batch-to-batch variation and the use of different bulk tRNAs limit the outcome of these analyses (Supplementary Figure S11C). Third, we produced several human Elongator variants that lack the regions important for tRNA or other subcomplex binding (i.e. CP2). In detail, we produced variants lacking (i) the tRNA binding loop of Elp1 (Elp1_Δ1132–1224_), (ii) the Elp1 binding loop of Elp4 (Elp4_Δ133–175_) and (iii) the very N-terminus of Elp5 (Elp5_Δ1–9_). The pull-down assays showed that all mutations moderately affect Elongator reconstitution, with stronger effects for Elp1_Δ1132–1224_ and Elp4_Δ133–175_ and less prominent reduction of binding by the Elp5 truncation (Figure 5C and Supplementary Figure S11D). These data indicated that the regions creating CP2 indeed affect the complex formation and that the loop region in Elp1, which was previously shown to contact the bound tRNA molecule is also involved in Elp456 recruitment. They also confirm a close link between tRNA binding and Elp456 recruitment. However, electrophoretic mobility shift assay (EMSA) showed that the Δ1132–1224 deletion in Elp1 displayed no significant effect on affinity of Elp123 variant toward mouse tRNA Ala^UGC^ compared to wild type subcomplex (Figure 5D). This is likely due to that tRNA binding seems to be dominated by Elp3 and the second contact site located in the Elp1 CTD might simply sense the presence of a tRNA or serves a role during specific transition states. This could explain our observation that the CTD loop deletion indeed displays functional importance *in vivo* even though there is no significant contribution to tRNA binding in the *in vitro* assay. It should also be noted that we were only able to test individual mutations and the observed interfaces are rather large. Hence, the remaining sites in a respective mutant (Elp1_Δ1132–1224_) might be sufficient to maintain tRNA binding. Finally, tRNA binding might not be affected by the tested mutants as the second Elp456-free lobe of Elp123 could mediate tRNA binding in the presence of Elp456. Nonetheless, the biochemical analyses of human Elongator support our structural findings, and several aspects need to be addressed by additional analyses in the future.

### High ATP concentration stimulates tRNA binding of Elp456

**Figure 6.**
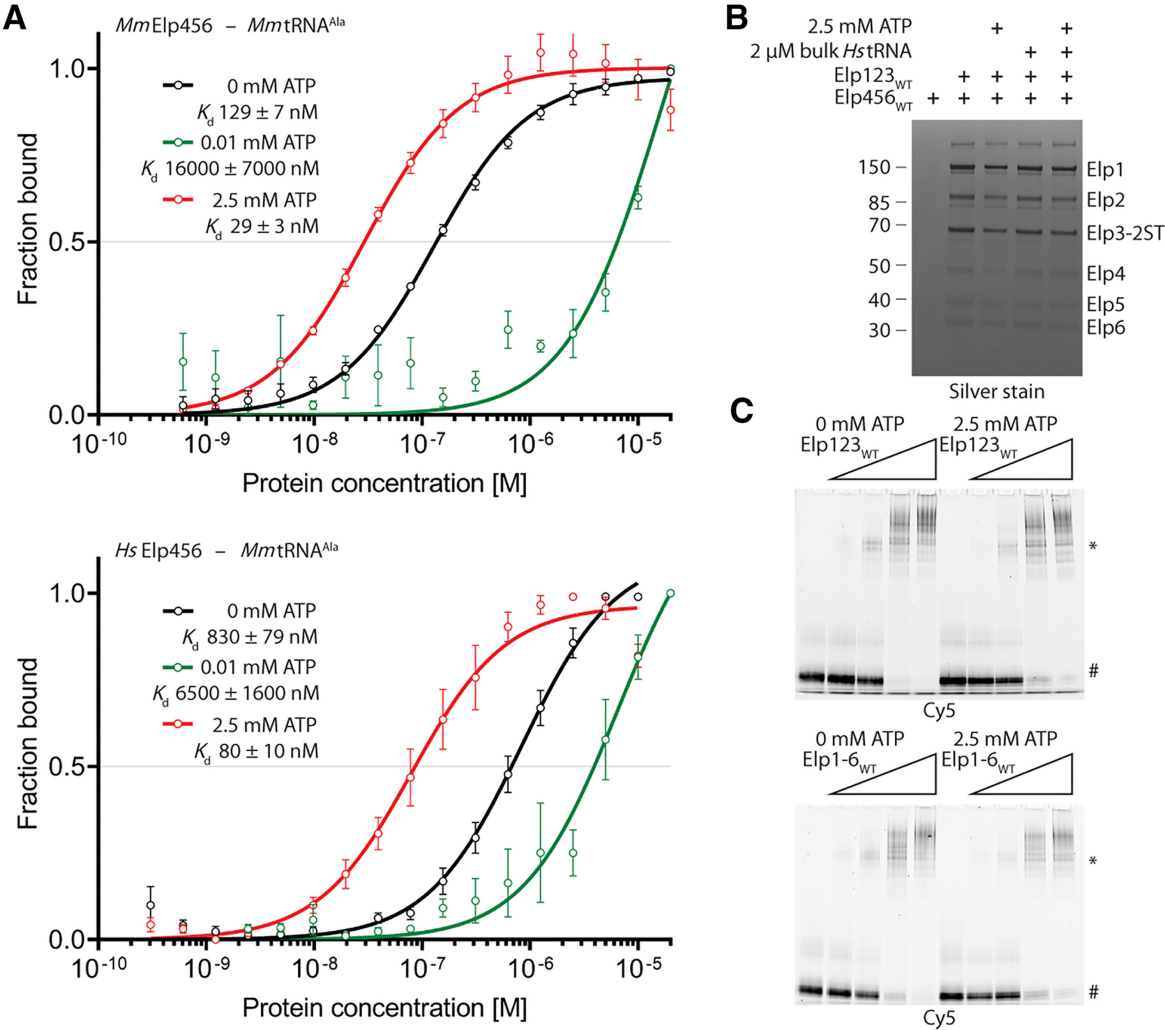
Biochemical and functional characterization of Elp456. (**A**) Microscale thermophoresis measurements, respective fits, and calculated dissociation constant (*K_d_*) values for mouse and human Elp456 at varying ATP concentrations. *n* = 3, *n* = 4 for mouse Elp456 and 0 mM ATP. (**B**) SDS-PAGE gel of *in vitro* reconstitution of human Elongator under varying ATP concentration and bulk tRNA presence. Twin-Strep-tag indicated on Elp3 subunit. (**C**) EMSA assay using fluorescently labeled tRNA Ala^UGC^ (0.2 μM), human Elp123 (top) and Elongator (bottom) (0.05–1 μM) in the absence and presence of ATP. The positions of free tRNA and protein–tRNA complexes (*) are indicated next to the native polyacrylamide gel electrophoresis.

**Figure 7.**
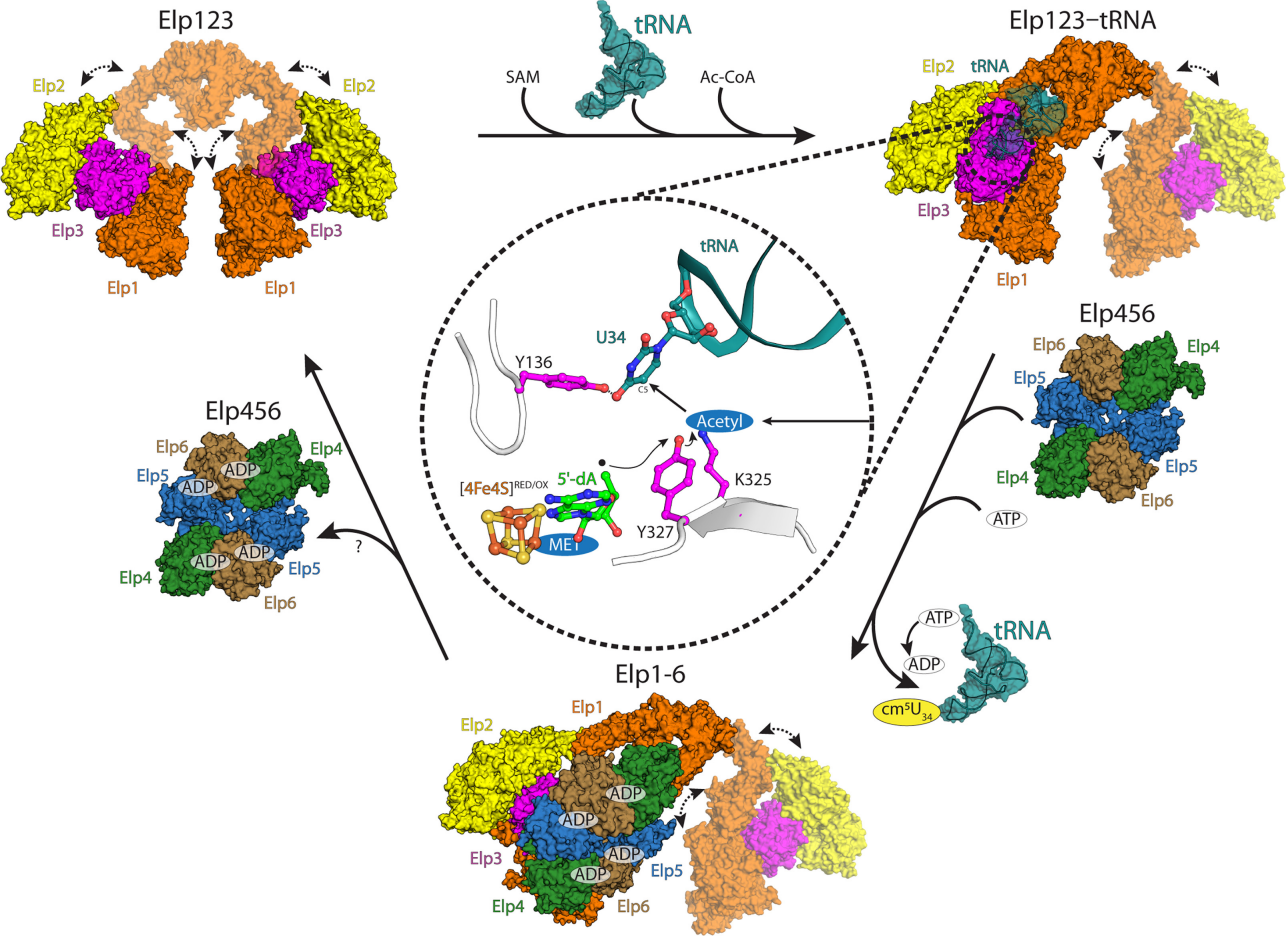
Elongator's reaction scheme. A schematic overview of the individual reaction intermediates of the eukaryotic Elongator complex. In higher mammalians highly flexible Elp123 binds tRNA substrate, SAM and Ac-CoA to modify U_34_. Bound tRNA substrate tethers the lobe with the CTD of Elp1. Next, SAM is cleaved to methionine and 5′-dA radical. The radical is passed on acetylated lysine with the help of tyrosine residue. Acetyl radical can then react with C5 of U_34_. After U_34_ modification, Elp456 assists in the release of certain tRNA species via Elp1 tRNA binding loop displacement. To allow tRNA binding to the Elp123 lobe occupied by Elp456 the smaller subcomplex needs to dissociate. How this occurs needs further investigation.

It was previously shown that yeast Elp456 binds tRNA in an ATP hydrolysis regulated manner (26). To verify whether mouse and human Elp456 behave in a similar fashion, we measured the binding affinity towards mouse tRNA Ala^UGC^ using MST assays with varying ATP concentrations (Figure 6A). Similar to yeast Elp456, low ATP concentration indeed decreases the affinity of both mammalian Elp456 complexes towards tRNA in comparison to samples without ATP. When physiologically relevant ATP concentrations (e.g. 2.5 mM) (99–101) were used, we detected a dramatic increase in binding affinities (Figure 6A). As our data show that Elp456 cannot only release tRNA upon hydrolysis of ATP but also bind strongly to tRNA at higher ATP concentration, we next investigated whether the interaction between human Elp123 and Elp456 could be ATP-dependent. The pull-down interaction assay showed no differences in the full complex assembly in the presence of various concentrations of ATP (Supplementary Figure S12A). We also performed the experiments for both mouse and human proteins in the presence of purified bulk tRNA to test if any minor tRNA species or tRNAs carrying modification intermediates may affect the reconstitution. Again, the full complex assembly is independent of tRNA, ATP or combinations thereof (Figure 6B and Supplementary Figure S12B, C). As the presence of ATP influences tRNA binding ability of Elp456, but not the assembly of the full complex, we further determined the impact of ATP on tRNA binding of the fully assembled reconstituted complex. The gel shift assay showed that the fully assembled human Elongator complex has slightly lower affinity towards tRNA in the presence of ATP (Figure 6C). The ATP effect on decrease of tRNA binding is also found in the Elp123. Based on this finding we would like to propose a molecular mechanism, in which Elp456 acts as a molecular extruder that binds tRNA with greater affinity and pulls it away from Elp123. A stronger Elp456–tRNA complex may fall apart upon successful ATP hydrolysis, releasing the tRNA and prepare Elp456 for another round of the reaction cycle, which in principle could promote the overall fidelity of tRNA modification. (Figure 7).

## DISCUSSION

tRNA anticodon modifications by Elongator have a broad implication for biomedicine and translational research, as its dysfunction is associated with a variety of severe human diseases. Yet, details of its mechanistic mode of action have remained elusive, leaving some of the most important questions related to this large macromolecular complex unanswered.

In this work, we developed novel protocols that allow overexpression and purification of the whole Elongator complex from yeast cells for the first time. Furthermore, we managed to overproduce, purify and reconstitute mouse and human Elongator complexes using advanced insect cell expression systems. We have determined several high-resolution cryo-EM structures for yeast and mouse Elongator complexes in the presence and absence of tRNAs. Foremost, the comparison of our cryo-EM structures demonstrates high similarities between yeast and mouse Elongator and their intermediates. Our results reveal how both subcomplexes interact with each other and that in both cases, the Elp456 ring binds to only one Elp123 lobe, leaving the second one unoccupied. As the reconstitutions of the mammalian Elongator complexes were carried out in excess of the smaller subcomplex the possibility existed that both Elp123 lobes would bind one Elp456 ring, resulting in a 1:2 stoichiometry of the subcomplexes. Yet, in our *Mm*Elongator structure the ring again binds only to one Elp123 lobe with a 1:1 stoichiometry, suggesting that the alternative scenario with two Elp456 rings binding both lobes of Elp123 at the same time appears rather unlikely. The poorly defined density of the second Elp123 lobe in both the *Sc*Elongator and *Mm*Elongator structures indicates its high flexibility, even in the presence of Elp456. Surprisingly, the CTD of Elp1 remains disordered in *Mm*Elp123. Nonetheless, binding of either tRNA or Elp456 do not cause major structural rearrangement but instead lead to a more compact and rigid complex. Therefore, the rather flexible mammalian Elongator complexes at the start of the reaction might undergo unanticipated structural rearrangements upon substrate binding, absent in yeast Elongator. It is noteworthy that we resolved a loop region (aa184–238) in yeast Elp1, which is essential for the modification activity *in vivo* and seems to support the integrity of the Elp123 subcomplex. As this loop is not visible in any of the previous cryo-EM structures and produces several violating crosslinks, it might become structured only under certain conditions indicating a dynamic component of complex assembly also for the yeast complex.

Apart from the overall structural similarities between fungal and murine Elongator, the spatial organization of their active sites is highly conserved as well. As the molecular mechanism of the tRNA modification reaction is still not fully characterized, we analyzed the conserved features of the various active sites in all our structures to understand the underlying principles. Selvadurai and colleagues proposed a catalytic reaction mechanism based on the biochemical characterizations of archaeal Elp3. The Elp3 utilizes its rSAM domain to accommodate and cleave SAM to produce a 5′-dA radical. The 5′-dA radical is then used to generate an acetyl radical where the acetyl group comes from the hydrolyzed ACO product or from a covalent acetyl-Elp3 intermediate (34). An unresolved issue is how the radical is transferred. The radical transfer may be similar to some ribonucleotide reductases, which are known to transfer generated radicals through a tyrosine relay system over a distance of ∼30 Å (102). Indeed, two structurally highly conserved tyrosine residues, namely Tyr136 and Tyr327, are present in yeast Elp3 active site, which are juxtaposed to the radical carrying methyl group of 5′-dA. For the full catalytic reaction to finish, it still requires the hydrolyzed acetyl group from the KAT domain to be transferred to the proposed Lys325 residue, which is residing in the rSAM domain. We speculate that Tyr327 might mediate the acetyl radical formation by bridging between the 5′-dA radical and the acetyl group (Figure 7). Of note, Tyr327 is crucial for Elongator's activity in yeast (31). After the creation of acetyl radical on Lys325, the C5 atom of U_34_ is ideally positioned to be modified, resulting in cm^5^U_34_. Moreover, our structures represent snapshots at certain stages of the catalytic reaction. First, the *Mm*Elp123 harbors a SAM molecule that is bound to the iron-sulfur cluster, indicating that SAM binding occurs before tRNA binding. Second, the *Sc*Elp123 in complex with tRNA harbors the SAM cleavage products, namely 5′-dA and methionine, in its catalytic site. Third, we could not identify any SAM molecule in the full Elongator, suggesting SAM is consumed. Hence, we depict the catalytic reaction as follows: (i) the Elp123 structure with intact SAM represents the initial state prior to tRNA binding and SAM cleavage; (ii) subsequently Elp123 mediates SAM cleavage upon tRNA binding (i.e. the Elp123–tRNA structure) which represents the stage of a radical generation just before its transfer from 5′-dA; (iii) the SAM free-Elongator depicts the post state of the catalytic reaction.

This is the first study to present the interaction of the Elp123 and Elp456 subcomplexes at high-resolution with sufficient structural details to provide pseudo-atomic models. Based on these structure analyses, we further biochemically characterized the role of Elp456 during the Elongator reaction cycle. It is interesting to see that the Elp456 ring occupies a similar region as the tRNA suggesting that Elp456 could spatially compete with a bound tRNA substrate. However, by super-positioning the structures of Elp123–tRNA and Elongator, we had to realize that tRNA still can spatially fit between Elp123 and Elp456. Despite the extensive efforts, we still could not manage to obtain any Elongator–tRNA structure. We further present that the CTD of Elp1 is essential for the catalytic activity and it has dual interacting targets, tRNA and Elp456. It seems that the interaction is discriminated to one or the other. However, the tRNA interaction with CTD is not crucial for tRNA recruitment.

tRNA binding to Elp123 is an independent event that does not require the presence of Elp456. However, the full Elongator complex displays different affinities to individual tRNA targets, suggesting a role for Elp456. The most obvious model, where binding of Elp456 directly competes with a bound tRNA molecule in Elp123, would have conveniently explained why we have not obtained a structure of the fully assembled complex with tRNA and that the full complex shows a decreased affinity for certain tRNAs. After comparing the structures of Elp123-tRNA and Elongator, we nevertheless had to realize that also the full complex would be able to accommodate a single tRNA molecule in the space formed between the Elp123 lobe and the Elp456 ring. Although, the tRNA binding loop in the CTD of Elp1 would not be able to interact with the elbow region of the bound tRNA, as it is displaced by the bound Elp456 ring. To gain further insights, we introduced specific mutations in human Elp1, which either remove the whole loop region or target specific regions of the loop (Δ1176–1200, Δ1201–1225 and Δ1226–1251). All three deletions *in vivo* in yeast Elp1 result in the inactivation of the Elongator pathway, but its removal in human Elp123 is not sufficient to diminish tRNA binding to Elp123, clearly proving that tRNA binding mainly occurs through the ASL bound in the active site of Elp3. The mechanistic rational behind tRNA selectivity and detailed intramolecular relay of tRNA remain elusive, but as Elp123 does not require Elp456 to bind tRNAs, we believe that Elp456 is important for the late stages of the modification reaction. Strikingly, we found that at physiologically relevant ATP concentration the affinity of mammalian Elp456 towards tRNAs exceeds the one measured for Elp123, respectively. As neither ATP nor tRNA influenced the interaction of Elp123 and Elp456, we speculate that ATP-binding might trigger the transition of an already modified tRNA to the Elp456 complex, which then releases the tRNA after ATP hydrolysis (Figure 7). As we were not able to detect any SAM molecule in the Elongator structures from mouse and yeast, we conclude that the Elp456-bound intermediate, represents the latest stage of the modification reaction and that after release from the Elp456 ring, the next reaction is initiated by binding of SAM, ACO and a modifiable tRNA.

Based on previous findings, we conclude that Elongator can bind all types of tRNAs, but only the recognition of specific bases in certain tRNAs triggers ACO hydrolysis in the KAT domain and SAM cleavage in the rSAM domain. In mammals, the binding of tRNAs induces large structural rearrangements in the arch domain, which might confer additional selectivity and create a second contact at the elbow region of the bound tRNA molecule. All modifiable tRNAs would receive cm^5^ at U_34_ by the activity of the Elp123, but some tRNAs additionally require the assistance of Elp456 to be unloaded from the complex after the modification reaction. To release certain modified tRNAs from Elp123, the smaller subcomplex would bind again and displace the loop in the Elp1 CTD allowing the tRNA to be released. We speculate that the affinity of certain tRNAs is simply too high for a release from Elp123 and that the displacement of the loop, facilitates the ATP-dependent transfer of tRNAs to the Elp456 ring. How the transfer from Elp123 to Elp456 exactly happens will require additional studies. In summary, our data provide novel insights into the structural and functional conservation of Elongator in eukaryotes and our work paves the way for further analyses of the intrinsic dynamics of Elongator and the specific differences between its tRNA substrate molecules.

## DATA AVAILABILITY

The models and cryo-EM densities map have been deposited in the EMData Bank (EMDB) and the Protein Data Bank (PDB) – yeast Elongator (PDB ID 8ASV and EMD-15622), mouse Elongator (EMD-15626), yeast Elp456 (PDB ID 8AT6 and EMD-15635), mouse Elp123 (PDB ID 8AVG and EMD-15682), yeast Elp123–tRNA (PDB ID 8ASW and EMD-15623), mouse Elp123–tRNA (EMD-15625). All XL-MS data are available at https://repository.jpostdb.org/entry/JPST001914 (ID JPST001914). All other data generated in this study are available from corresponding authors on reasonable request.

## Supplementary Material

gkac1232_Supplemental_File

## Notes

Present address: Rościsław Krutyhołowa, Department of Biology, Institute of Molecular Biology and Biophysics, ETH Zurich, Zurich 8093, Switzerland.
