# Supplementary material for: Cryo-EM structure of the fully assembled Elongator complex
Source: Nucleic Acids Res. 2023 Jan 9;51(5):2011–32. doi: 10.1093/nar/gkac1232 (PMC10018365; doi:10.1093/nar/gkac1232)
Supplement: gkac1232_Supplemental_File [file gkac1232_supplemental_file.pdf]

Supplementary Information

Cryo-EM structure of the fully assembled Elongator complex

Marcin Jaciuk[1], David Scherf[2], Karol Kaszuba[3,4], Monika Gaik[1], Alexander Rau[5], Anna Koscielniak[1], Rościsław Krutyhołowa[1], Michal Rawski[1,6], Paulina Indyka[1,6], Andrea Graziadei[5], Andrzej Chramiec-Głąbik[1], Anna Biela[1], Dominika Dobosz[1], Ting-Yu Lin[1], Nour-el-Hana Abbassi[1,7], Alexander Hammermeister[1,2], Juri Rappsilber[5,8], Jan Kosinski[3,4,9], Raffael Schaffrath[2] and Sebastian Glatt[1,*]

[1] Małopolska Centre of Biotechnology (MCB), Jagiellonian University, Krakow, 30-387, Poland
[2] Institute for Biology, Department for Microbiology, University of Kassel, Kassel, 34132, Germany
[3] European Molecular Biology Laboratory Hamburg, Hamburg, 22607, Germany.
[4] Centre for Structural Systems Biology (CSSB), Hamburg, 22607, Germany.
[5] Bioanalytics, Institute of Biotechnology, Technische Universität Berlin, Berlin, 13355, Germany
[6] National Synchrotron Radiation Centre SOLARIS, Jagiellonian University, Krakow, 30-387, Poland
[7] Postgraduate School of Molecular Medicine, Medical University of Warsaw, 02-091, Poland
[8] Wellcome Centre for Cell Biology, University of Edinburgh, Edinburgh EH9 3BF, UK
[9] Structural and Computational Biology Unit, European Molecular Biology Laboratory, Heidelberg, 69117, Germany.

* To whom correspondence should be addressed. Tel: +48 12 664 6321 Fax: +48 12 664 6902; Email: sebastian.glatt@uj.edu.pl

Present Address: Rościsław Krutyhołowa, Department of Biology, Institute of Molecular Biology and Biophysics, ETH Zurich, Zurich, 8093, Switzerland

Supplementary Figures

Figure S1. Recombinant *Sc*Elongator purification.

Figure S2. *Sc*Elongator cryo-EM reconstruction.

Figure S3. *Sc*Elp456 cryo-EM reconstructions.

Figure S4. Phenotypic analyses of yeast strains carrying mutations in selected subunits.

Figure S5. Further *Sc*Elongator model validation.

Figure S6. Purification of mammalian Elongator subcomplexes.

Figure S7. *Mm*Elp123 alone and in complex with tRNA cryo-EM reconstructions.

Figure S8. *Sc*Elp123 in complex with tRNA cryo-EM reconstruction.

Figure S9. tRNA binding comparison between yeast and mouse Elp123 subcomplexes.

Figure S10. *Mm*Elongator cryo-EM reconstruction.

Figure S11. Mammalian Elongator reconstitution, biochemical and mutational analyses.

Figure S12. Mammalian Elongator reconstitution is not affected by ATP or tRNA presence.

Table S1. Used yeast strains.

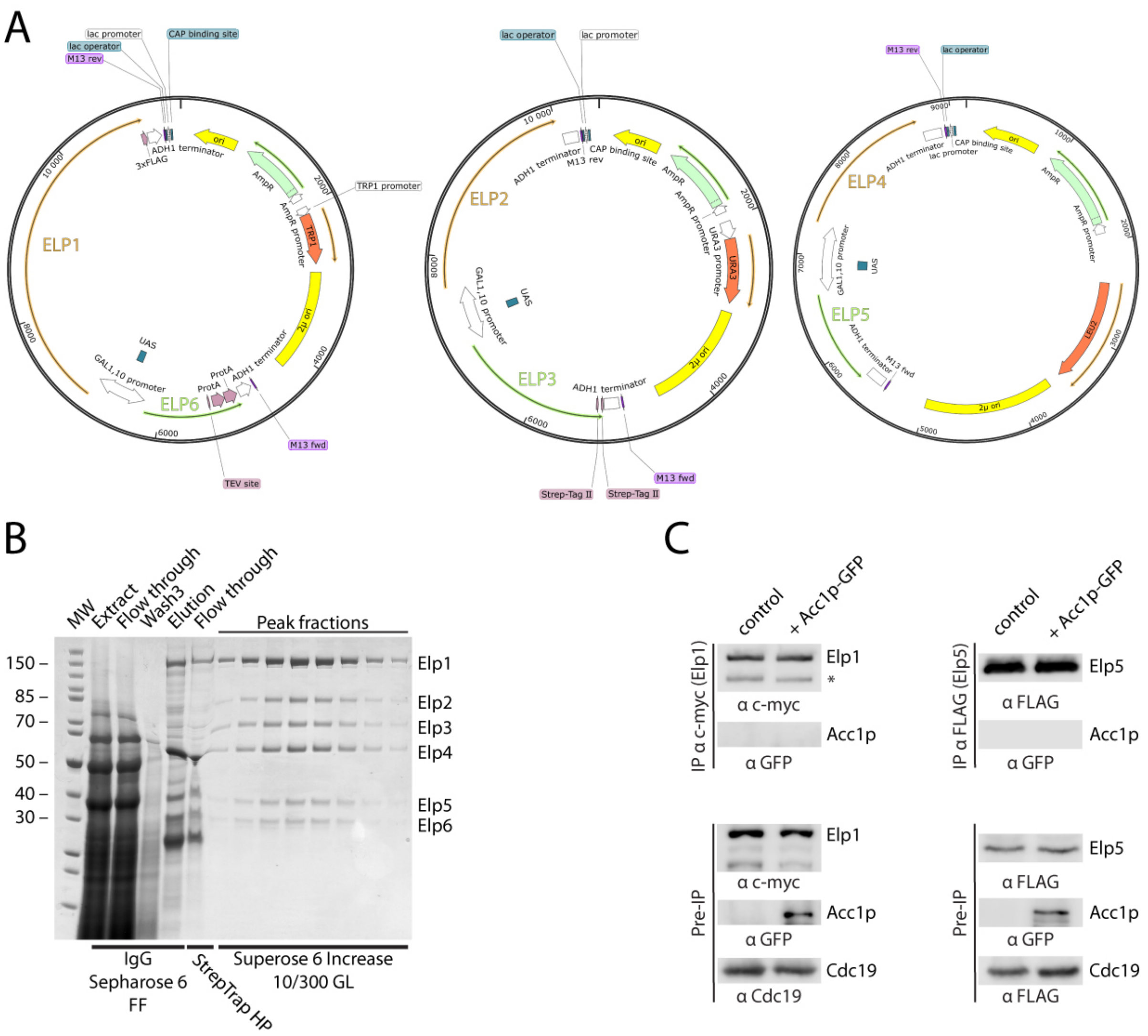

Figure S1. Recombinant *Sc*Elongator purification. (A) Maps of plasmids used for overproduction of yeast Elongator in yeast cell cultures. (B) SDS-PAGE gel with fractions from full yeast Elongator purification process. (C) Immunoprecipitation (IP) study to test for copurification of Acc1 protein with endogenous yeast Elongator subunit Elp1 and Elp5. Asterisk denotes characteristic N-terminal proteolysis signal of Elp1 (1).

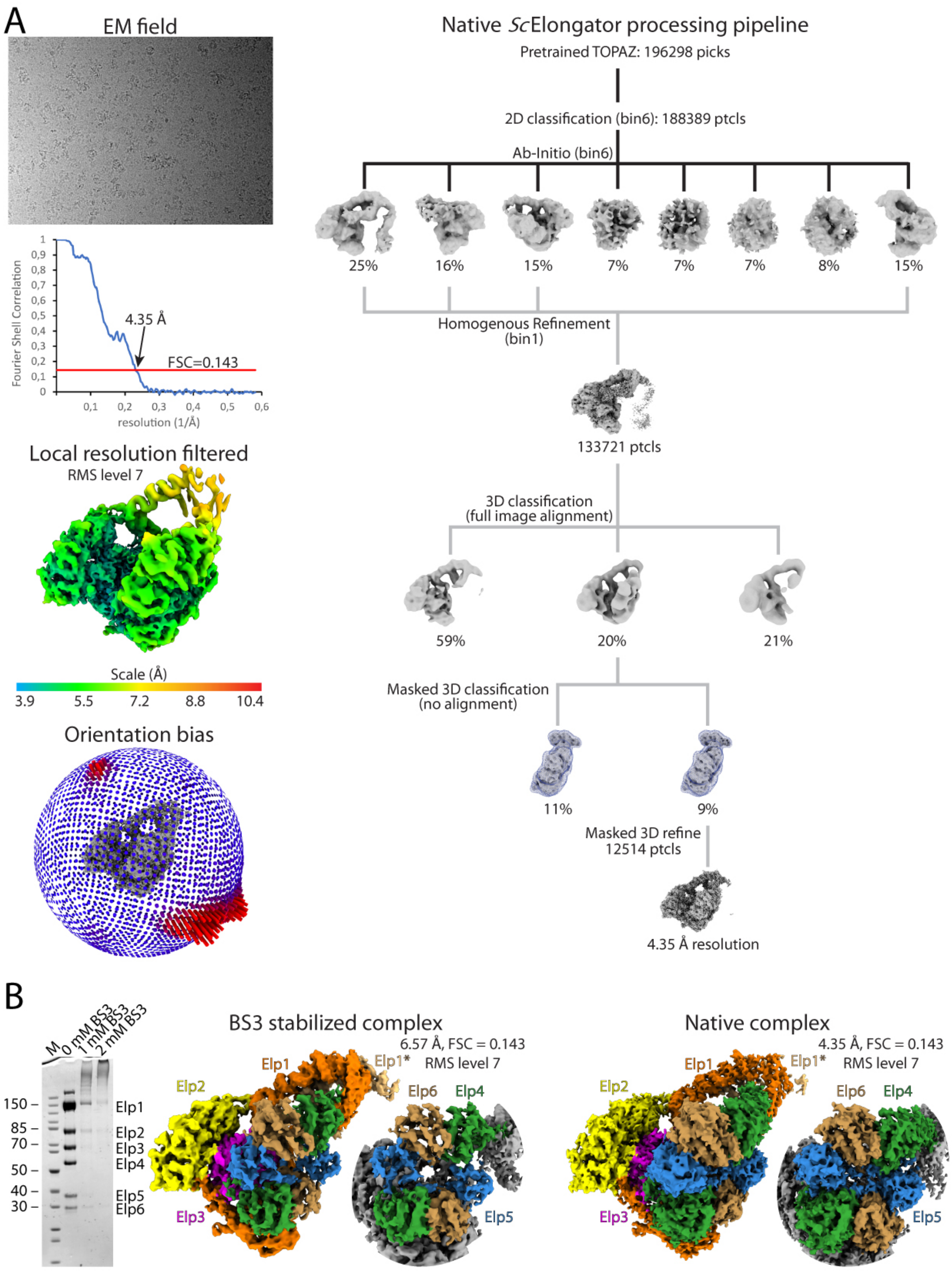

Figure S2. *Sc*Elongator cryo-EM reconstruction. (A) Representative cryo-EM field of yeast Elongator complex. General processing pipeline. Fourier Shell Correlation curve for yeast Elongator. The red line indicates FSC=0.143 with the labelled resolution cutoff. The resolution range of yeast Elongator map displayed on the local resolution filtered map visualized at indicated map contouring. Particle orientation bias of yeast Elongator map. (B) SDS-PAGE showing BS3 stabilized yeast Elongator complex and cryo-EM maps comparing the stabilized and native complex. Front view map with coloring according to the scheme from Figure 1D. Only the density, corresponding to the smaller subcomplex, is colored in the Elp456 view.

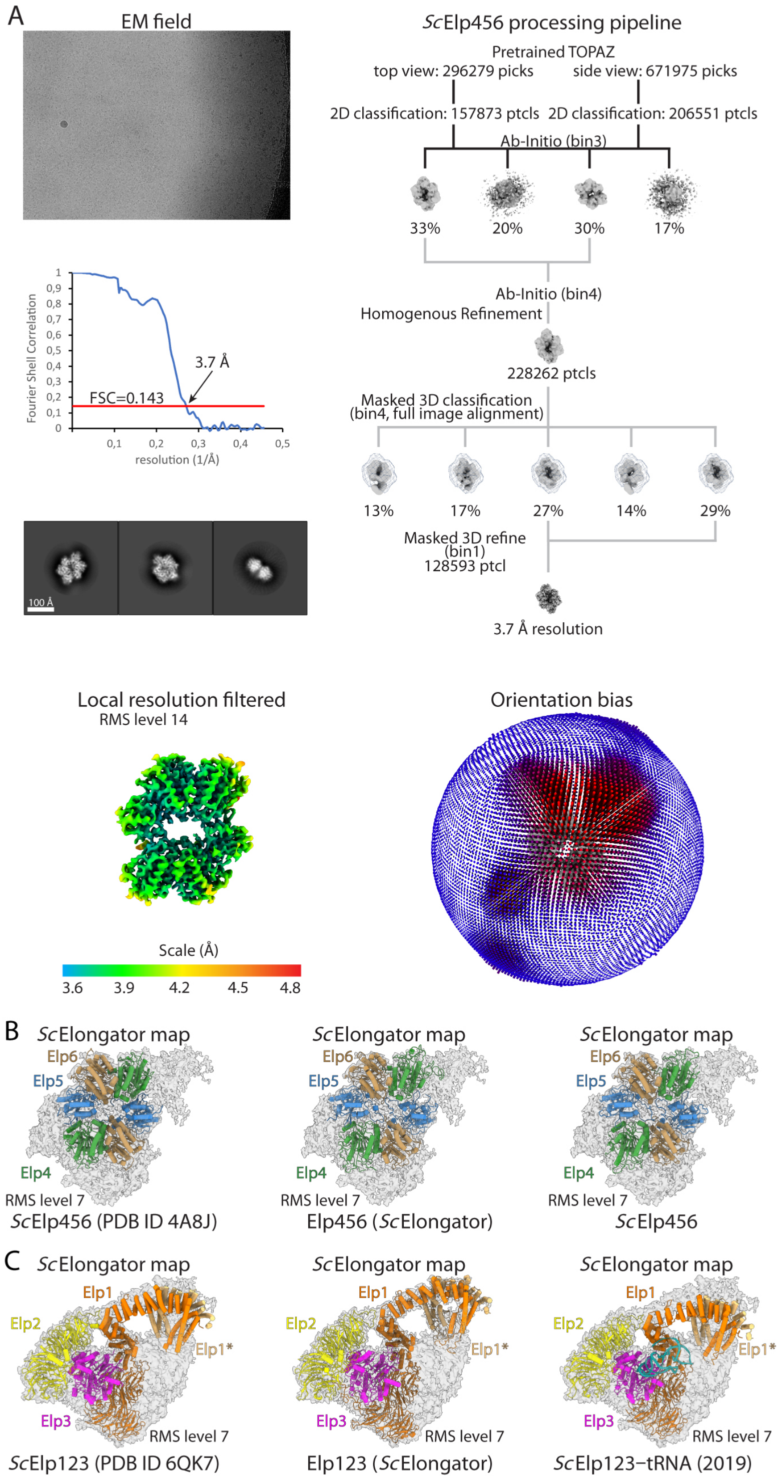

Figure S3. *Sc*Elp456 cryo-EM reconstructions. (A) Representative cryo-EM field and general processing pipeline of *Sc*Elp456 subcomplex. Fourier Shell Correlation curve. The red line indicates FSC=0.143 with the labelled resolution cutoff. Reference-free 2D class averages, scale bar 100 Å. The resolution range displayed on the local resolution filtered map visualized at indicated map contouring. Particle orientation bias. (B) Comparison of yeast crystal and cryo-EM apo Elp456 with the one from Elongator structure. Models were fit into cryo-EM density of yeast Elongator and visualized at indicated contouring. (C) Comparison of yeast cryo-EM free and tRNA bound Elp123 with the one from Elongator structure. Models were fit into cryo-EM density of yeast Elongator and visualized at indicated contouring.

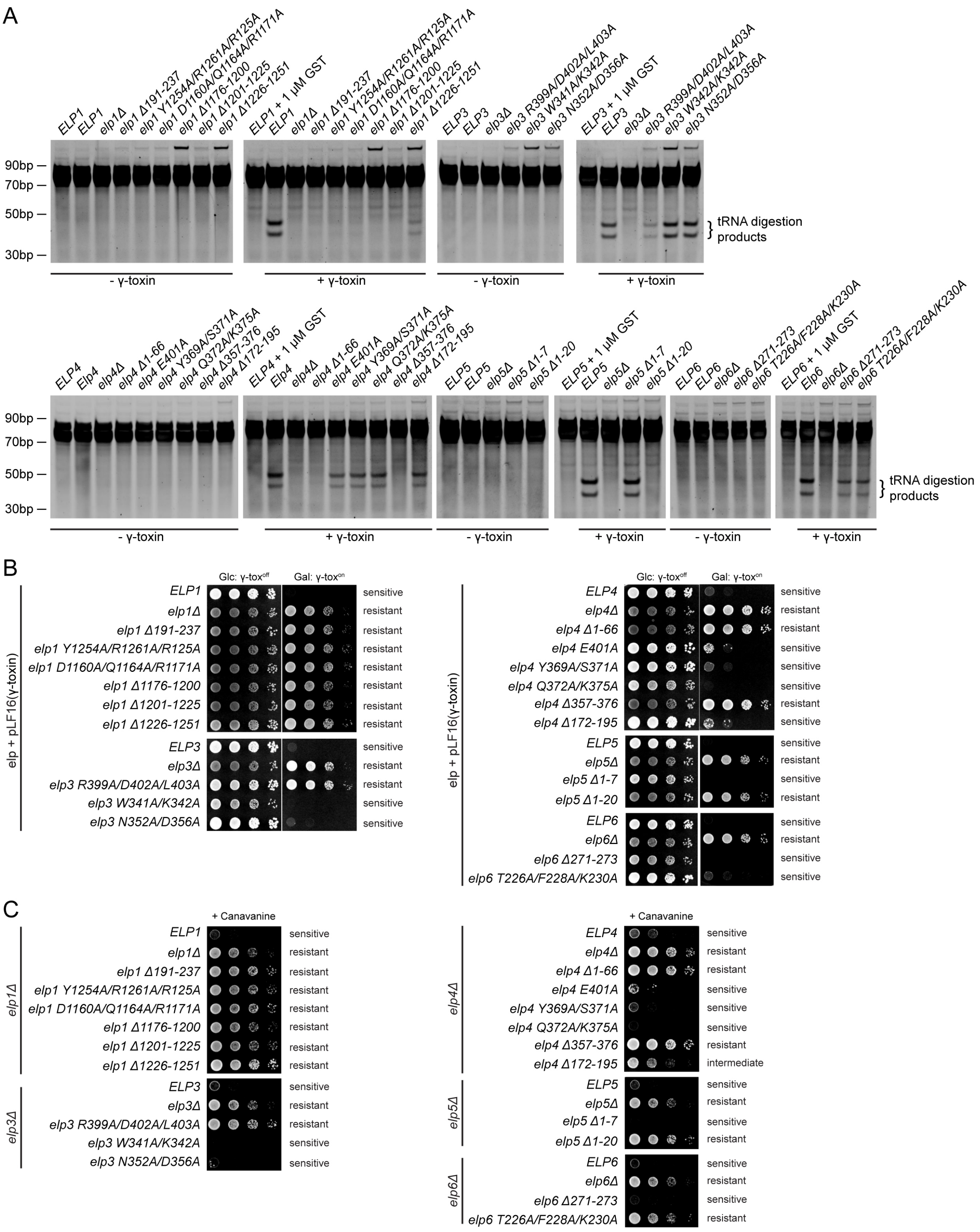

Figure S4. Phenotypic analyses of yeast strains carrying mutations in selected subunits. (A) TBE-UREA gels showing *in vitro* γ-toxin cleavage of isolated yeast bulk tRNA. Mutations disturbing Elongator activity render yeast cells insensitive to anticodon cleavage by γ-toxin. (B) Endogenous γ-toxin assay. Indicated yeast strains were transformed with the vector pLF16 carrying the γ-toxin gene under the galactose-inducible *GAL1* promoter. Ten-fold serial cell dilutions were spotted on SD media containing 2% glucose (Glc: γ-tox^{off}) or 2%galactose (Gal: γ-tox^{on}) and were incubated for 3-4 days at 30°C. Mutations disturbing Elongator activity render yeast cells resistant to growth inhibition by γ-toxin. (C) Canavanine assay. Toxic arginine analog uptake from the environment prevents yeast cells from growing. For more information, see the main text and Materials and Methods section.

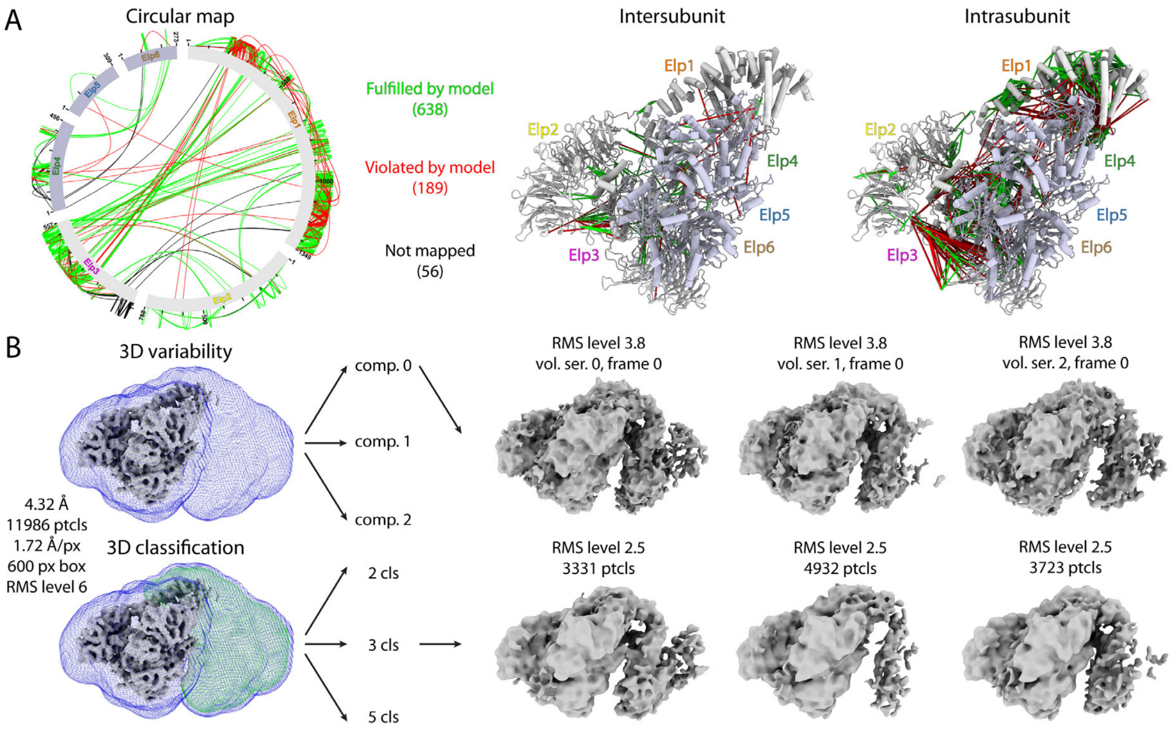

Figure S5. Further *Sc*Elongator model validation. (A) Crosslinking mass spectrometry of yeast Elongator. On the left circular map representing all detected cross links, followed by yeast Elongator model with mapped intersubunit (left) and intrasubunit (right) crosslinks. (B) Additional 3D variability (top) and 3D classification (bottom) analyses to resolve density of the second lobe. In blue colour mask designed on BS3 stabilized Elongator reconstruction (see Figure 1D), in green focus mask used during 3D classification.

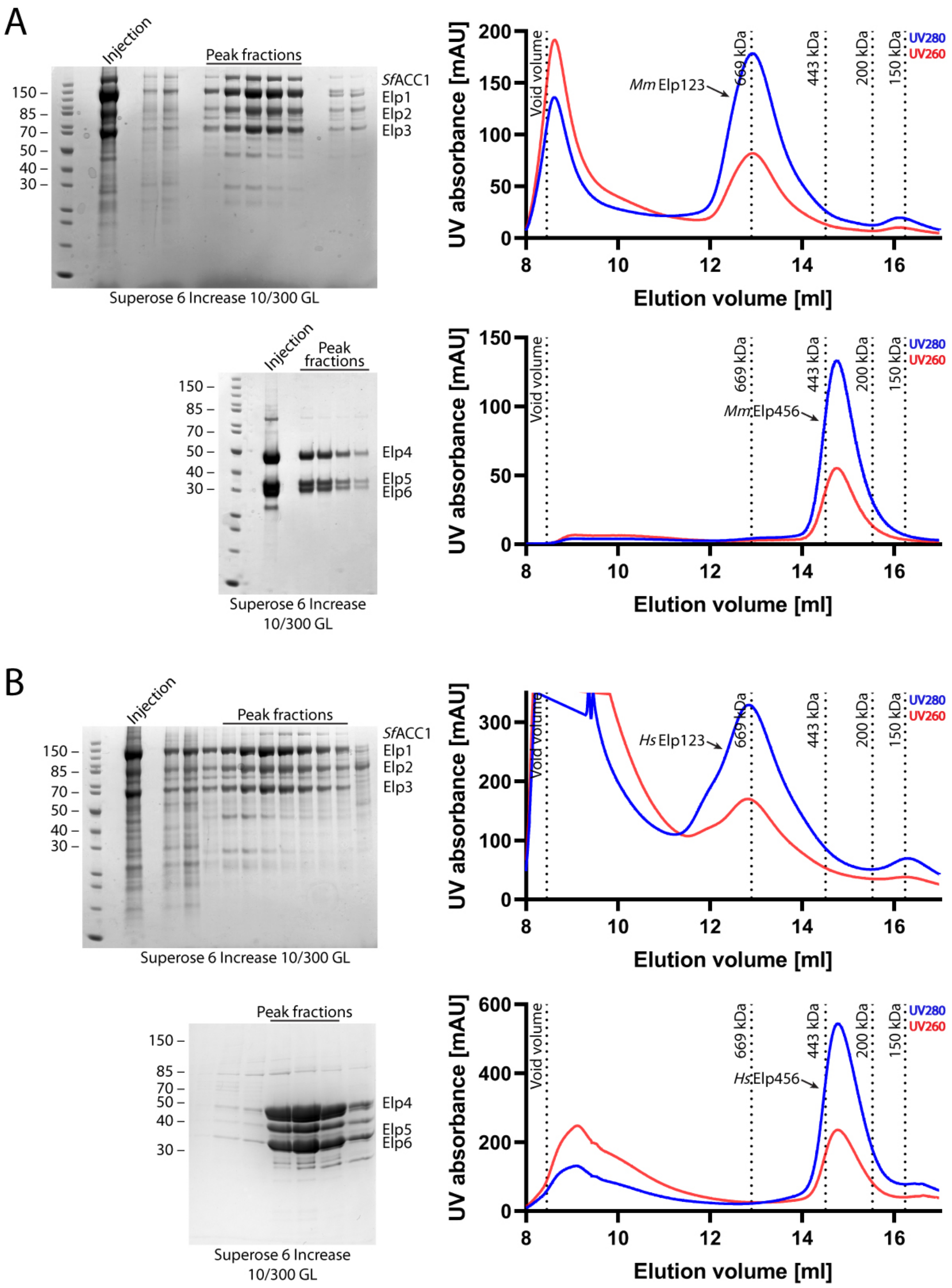

Figure S6. Purification of mammalian Elongator subcomplexes. SDS-PAGE gels with fractions from the last step of the purification process and corresponding size exclusion chromatography profiles. Red and blue lines represent UV absorption at 260 and 280 nm respectively. Peaks corresponding to specific subcomplexes are indicated with a black arrow. (A) mouse and (B) human Elp123 and Elp456, respectively.

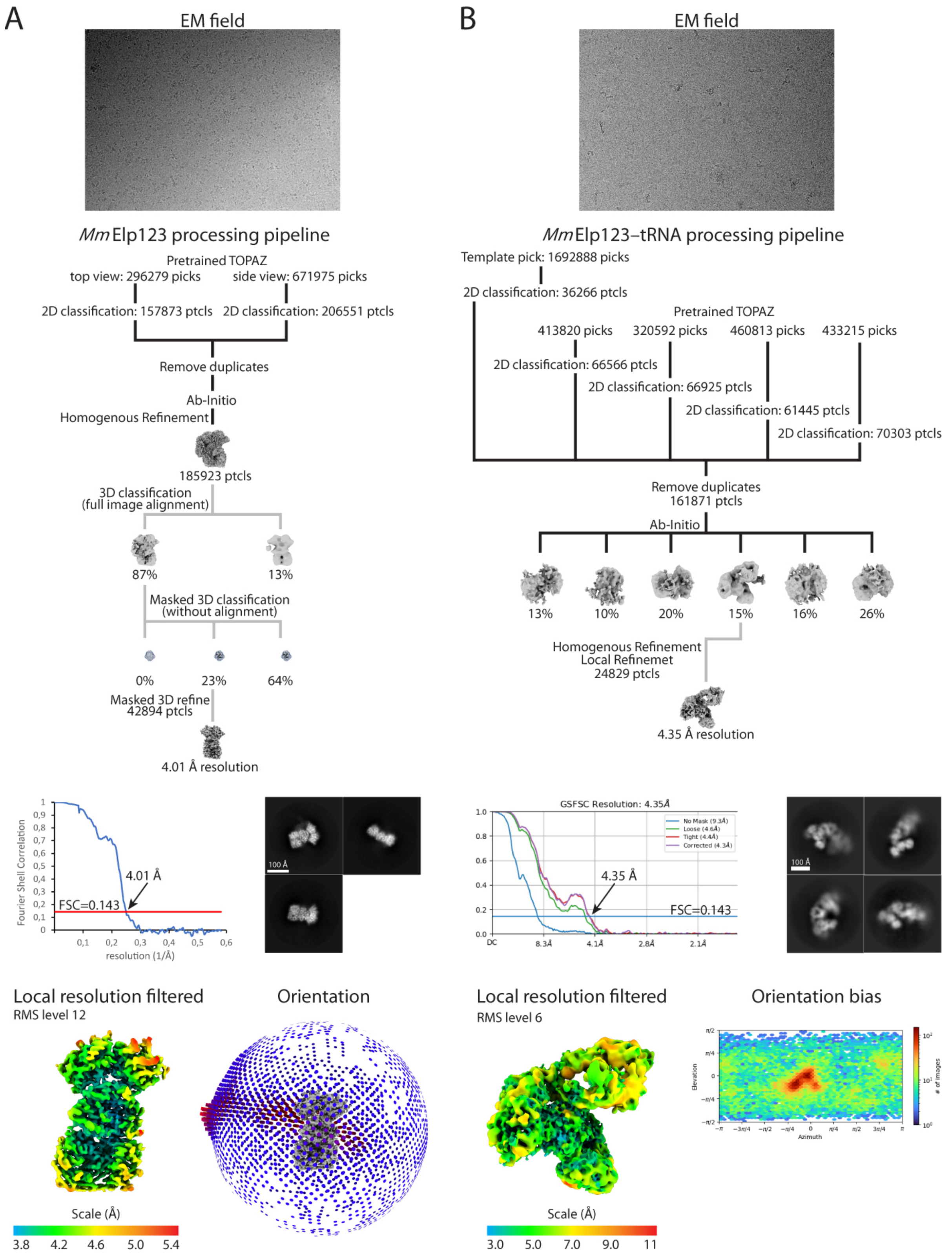

Figure S7. Cryo-EM reconstructions of *Mm*Elp123 alone and in complex with tRNA. General processing pipeline and representative cryo-EM field of (A) *Mm*Elp123 and (B) *Mm*Elp123–tRNA. The red line in the Fourier Shell Correlation curve indicates a FSC=0.143 – the respective resolution cutoff is indicated. In addition, selected reference-free 2D class averages are shown (scale bar 100 Å). At the bottom, the resolution range is displayed on the local resolution filtered map (visualized at indicated map contouring levels) and the orientation bias of the particle sets are shown.

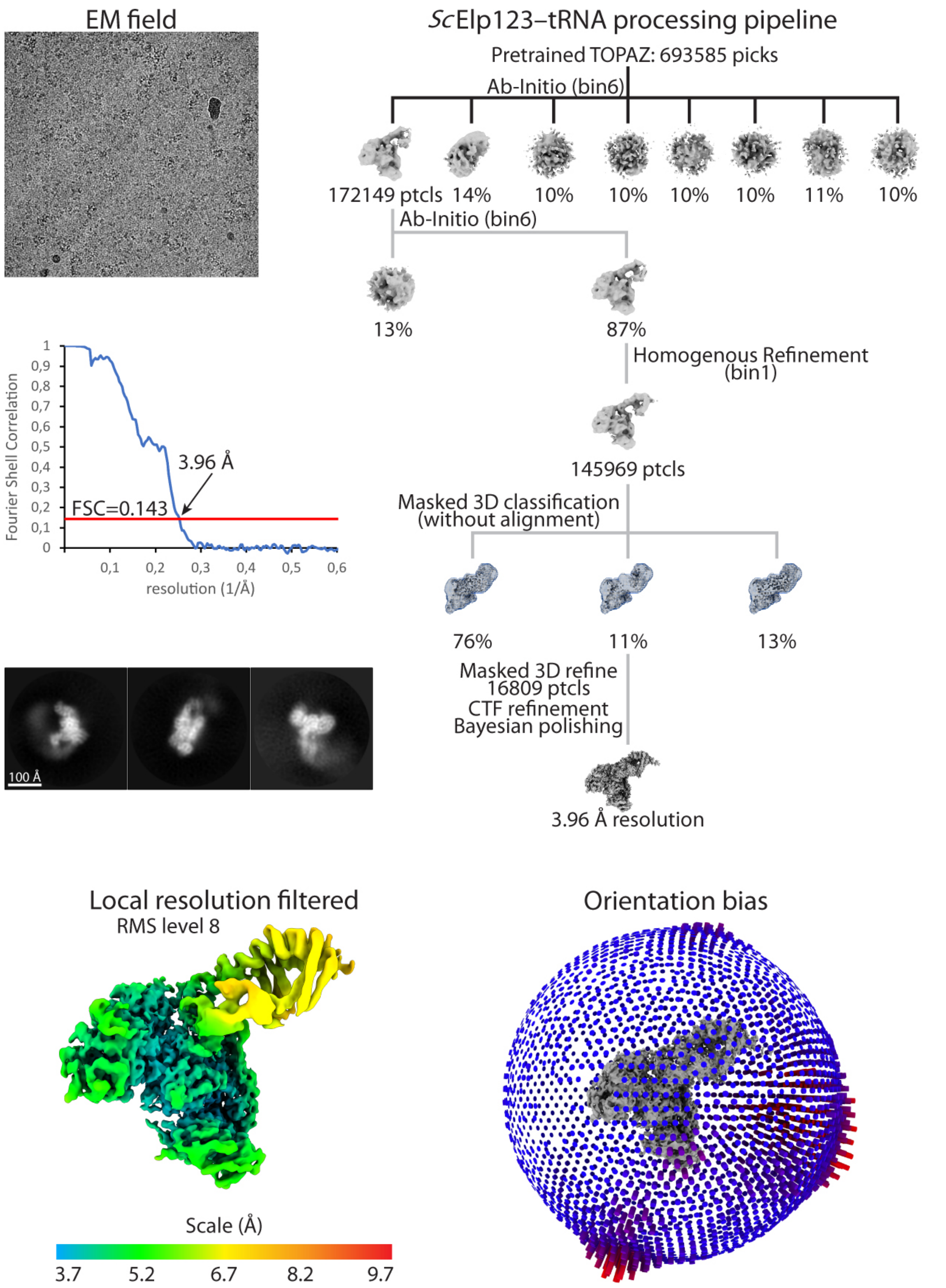

Figure S8. *Sc*Elp123 in complex with tRNA cryo-EM reconstruction. Representative cryo-EM field and general processing pipeline of *Sc*Elp123–tRNA complex. Fourier Shell Correlation curve. The red line in the Fourier Shell Correlation curve indicates a FSC=0.143 – the respective resolution cutoff is indicated. In addition, selected reference-free 2D class averages are shown (scale bar 100 Å). At the bottom, the resolution range is displayed on the local resolution filtered map (visualized at indicated map contouring levels) and the orientation bias of the particle sets are shown.

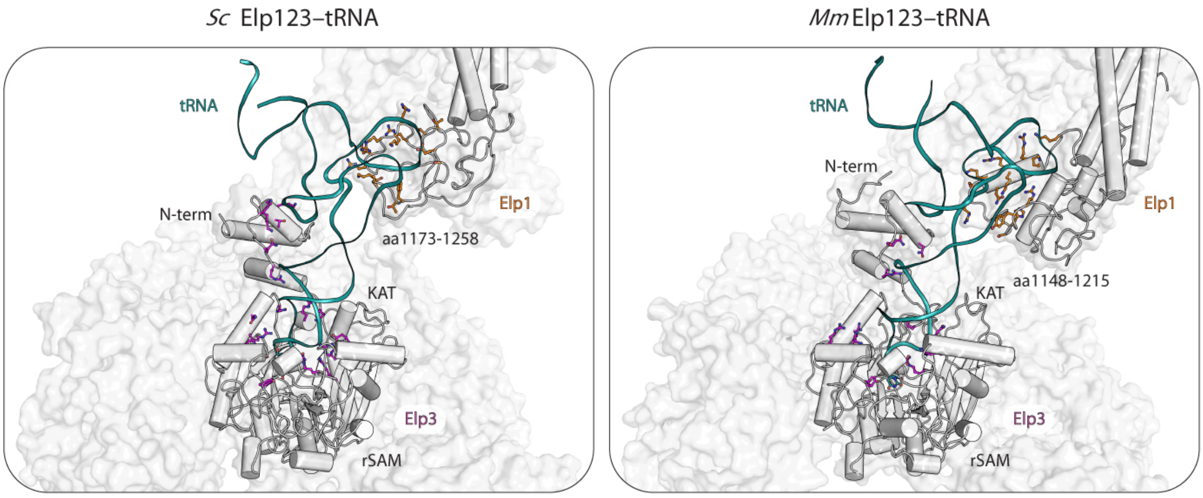

Figure S9. tRNA binding comparison between yeast and mouse Elp123 subcomplexes. Elp3 and Elp1 C-terminus are shown with distinguished residues that take part in the interaction with the tRNA molecules.

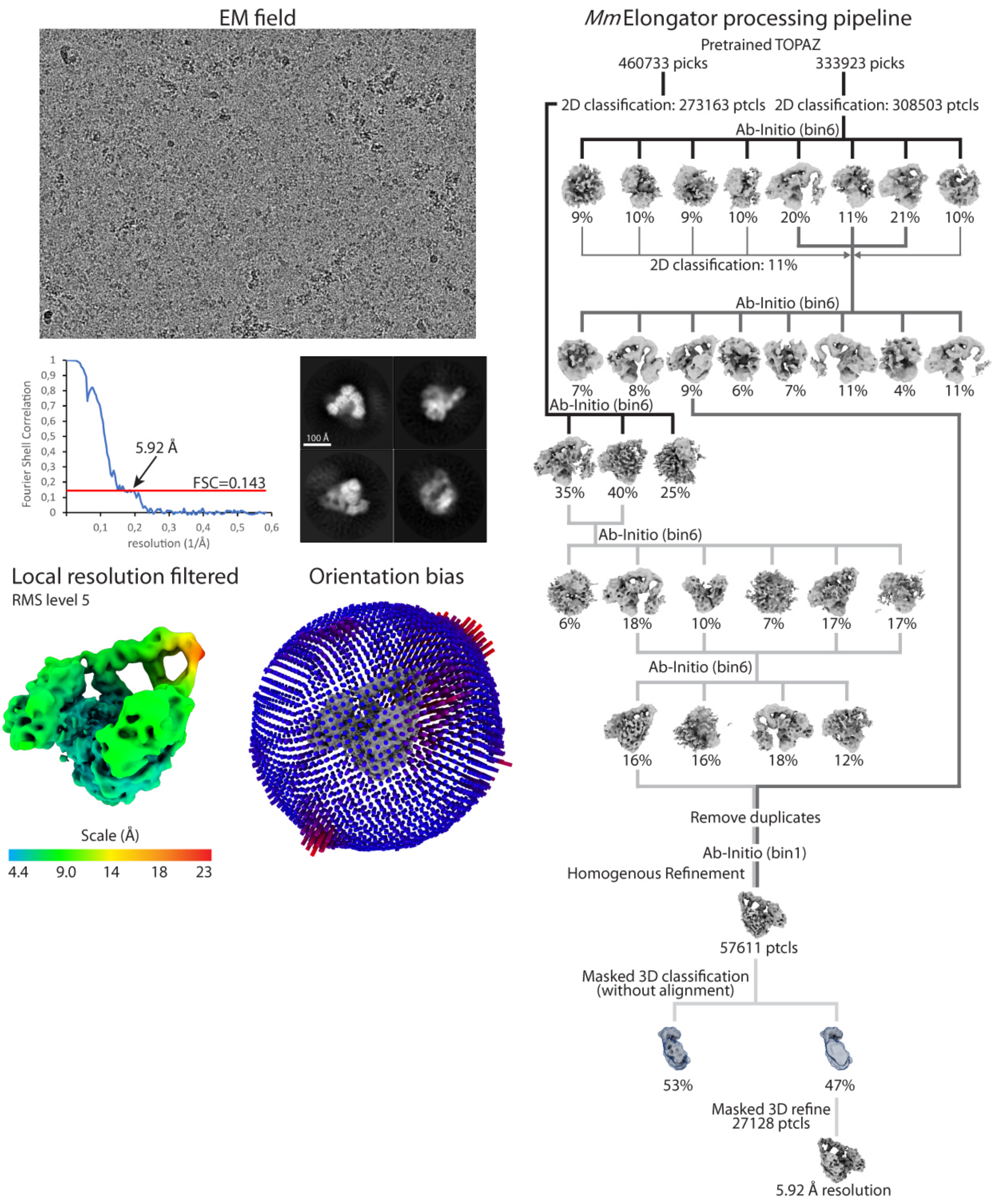

Figure S10. *Mm*Elongator cryo-EM reconstruction. Representative cryo-EM field and general processing pipeline of *Mm*Elongator. Fourier Shell Correlation curve. The red line in the Fourier Shell Correlation curve indicates a FSC=0.143 – the respective resolution cutoff is indicated. In addition, selected reference-free 2D class averages are shown (scale bar 100 Å). At the bottom, the resolution range is displayed on the local resolution filtered map (visualized at indicated map contouring levels) and the orientation bias of the particle sets are shown.

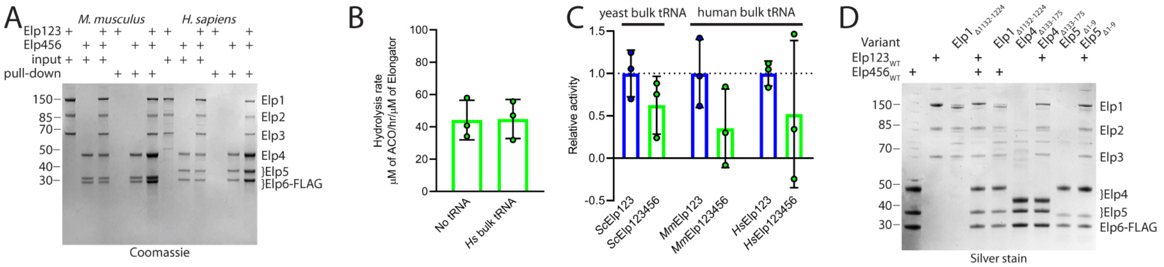

Figure S11. Mammalian Elongator reconstitution, biochemical and mutational analyses. (A) SDS-PAGE analysis shows, the FLAG pull-down assay of *in vitro* reconstructed mouse and human Elongator complex, via FLAG-tagged Elp6 subunit. FLAG-tag indicated on Elp6 subunit. (B) Mean acetyl-CoA hydrolysis rate of yeast Elongator (green) shows no stimulation of Elongator activity using human bulk tRNA. n = 3. (C) Mean relative acetyl-CoA activity of Elp123 (blue) and Elongator (green) from yeast, mouse and human in presence of yeast and human bulk tRNA. n = 3 (D) Sample inputs for FLAG pull-down assays from Figure 5C. FLAG-tag indicated on Elp6 subunit.

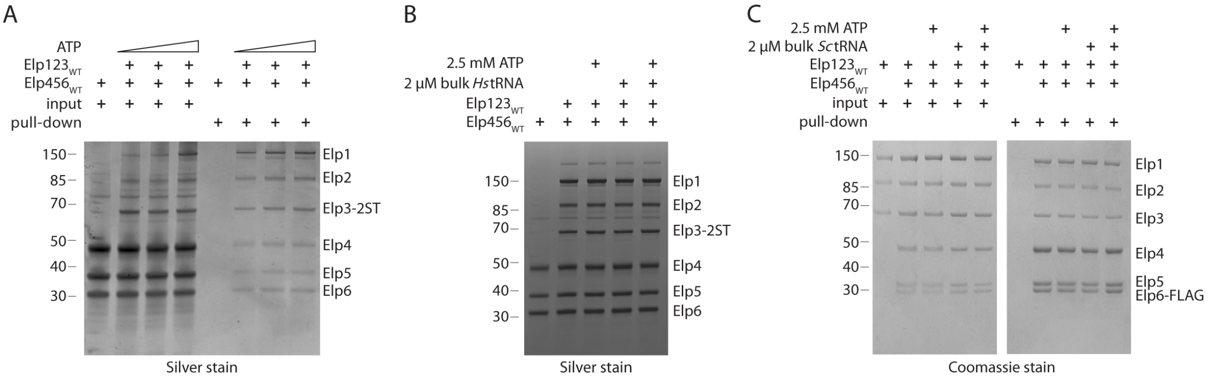

Figure S12. Mammalian Elongator reconstitution is not affected by ATP or tRNA presence. (A) SDS-PAGE analysis of *in vitro* reconstitution of human Elongator in presence of varying ATP concentrations. Twin-Strep-tagged Elp3 subunit (Elp3-2ST) was used in the pull-down assay. (B) Sample inputs for the Elp3 Twin-Strep (Elp3-2ST) pull-down assays from Figure 6B. (C) Elp6-FLAG pull-down showing the *in vitro* reconstruction of mouse Elongator using2.5 mM ATP and 2 μM bulk yeast tRNA.

Table S1. Used yeast strains.

Strain	Relevant genotype	Source
UMY2893	MATα *SUP4 leu2-3,112 trp1-1 can1-100 ura3-1 ade2-1 his3-11,15*	(2)
ySF88	UMY2893, *elp1Δ::KlURA3*	This study
ySF90	UMY2893, *elp3Δ::KlURA3*	This study
ySF91	UMY2893, *elp4Δ::KlURA3*	This study
ySF92	UMY2893, *elp5Δ::KlURA3*	This study
ySF93	UMY2893, *elp6Δ::KlURA3*	This study
ySF135	ySF88, *elp1_Δ191-237::KlTRP1*	This study
ySF136	ySF88, *elp1_Y1254A/R1261A/R1265A::KlTRP1*	This study
ySF137	ySF88, *elp1_D1160A/Q1164A/R1171A::KlTRP1*	This study
ySF138	ySF88, *elp1_Δ1176-1200::KlTRP1*	This study
ySF139	ySF88, *elp1_Δ1201-1225::KlTRP1*	This study
ySF140	ySF88, *elp1_Δ1226-1251::KlTRP1*	This study
ySF141	ySF90, *elp3_R399A/D402A/L403A::KlTRP1*	This study
ySF142	ySF90, *elp3_W341A/K342A::KlTRP1*	This study
ySF143	ySF90, *elp3_N352A/D356A::KlTRP1*	This study
ySF144	ySF91, *elp4_Δ1-66::KlTRP1*	This study
ySF145	ySF91, *elp4_E401A::KlTRP1*	This study
ySF146	ySF91, *elp4_Y369A/S371A::KlTRP1*	This study
ySF147	ySF91, *elp4_Q372A/K375A::KlTRP1*	This study
ySF148	ySF91, *elp4_Δ357-376::KlTRP1*	This study
ySF149	ySF91, *elp4_Δ172-195::KlTRP1*	This study
ySF150	ySF92, *elp5_Δ1-7::KlTRP1*	This study
ySF151	ySF92, *elp5_Δ1-20::KlTRP1*	This study
ySF152	ySF93, *elp6_Δ271-273::KlTRP1*	This study
ySF153	ySF93, *elp6_T226A/F228A/K230A::KlTRP1*	This study
BY4741	MATα *his3Δ1 leu2Δ0 ura3Δ0met15Δ0*	Euroscarf
ATCC 201388	MATα *his3Δ1 leu2Δ0 met15Δ0 ura3Δ0*	(3)
ySF312	BY4741, *ELP1-(c-myc)$_{3x}$::KlLEU2*	This study
ySF313	ATCC 201388, *ACC1-GFP::HIS3MX6, ELP1-(c-myc)$_{3x}$::clonNAT*	This study
ySF314	BY4741, *ELP5-(FLAG)$_{3x}$::KlLEU2*	This study
ySF315	ATCC 201388, *ACC1-GFP::HIS3MX6, ELP5-(FLAG)$_{3x}$::KlLEU2*	This study

References

1. Fichtner,L., Jablonowski,D., Schierhorn,A., Kitamoto,H.K., Stark,M.J. and Schaffrath,R. (2003) Elongator's toxin-target (TOT) function is nuclear localization sequence dependent and suppressed by post-translational modification. *Mol Microbiol*, **49**, 1297–1307.

2. Huang,B., Johansson,M.J.O., Byström,A.S., Bystrom,A.S., Byström,A.S. and Bystrom,A.S. (2005) An early step in wobble uridine tRNA modification requires the Elongator complex. *RNA*, **11**, 424–436.

3. Huh,W.K., Falvo,J. V., Gerke,L.C., Carroll,A.S., Howson,R.W., Weissman,J.S. and O'Shea,E.K. (2003) Global analysis of protein localization in budding yeast. *Nature*, **425**, 686–691.
